# Direct Modeling of DNA and RNA Aptamers with AlphaFold 3: A Promising Tool for Predicting Aptamer Structures and Aptamer–Target Interactions

**DOI:** 10.1021/acssynbio.5c00196

**Published:** 2025-07-03

**Authors:** Steven Ochoa, Valeria Tohver Milam

**Affiliations:** † School of Materials Science and Engineering, 1372 Georgia Institute of Technology, 771 Ferst Dr. NW, Atlanta, Georgia 30332-0245, United States; ‡ School of Materials Science and Engineering, Parker H. Petit Institute for Bioengineering and Bioscience, Georgia Institute of Technology, 771 Ferst Dr. NW, Atlanta, Georgia 30332-0245, United States

**Keywords:** canonical, ligand, noncanonical, oligonucleotide, secondary structure prediction, structure-binding, Protein Data Bank

## Abstract

Aptamers, single-stranded nucleic acids that fold into unique three-dimensional shapes, bind selectively to non-nucleotide target molecules, making them promising ligands for diagnostic and therapeutic applications. The ability to accurately predict folded aptamer structures and their molecular interactions would significantly enhance the rational design of nucleic acid-based affinity reagents. However, predicting the 3D structures of aptamers remains challenging due to their complex folding patterns and limited experimental structure data compared to proteins. AlphaFold 3 is the latest structure prediction tool by Google DeepMind that has recently expanded to include nucleic acids and small molecule targets, offering new possibilities for the direct 3D modeling of aptamer sequences. This study evaluates the accuracy of AlphaFold 3 by comparing its predictions to experimentally resolved aptamer structures in the Protein Data Bank (PDB) and to well-characterized aptamers not included in the PDB. AlphaFold 3 effectively modeled a range of PDB-resolved aptamer structures, including those with noncanonical secondary structure elements such as G-quadruplexes and pseudoknots. For non-PDB aptamers, AlphaFold predictions were considerably less confident yet showed reasonable overlap with experimental data, accurately predicting G-quadruplex conformations and, in some cases, correctly localizing known binding interfaces in aptamer–protein complexes. Despite these attributes, AlphaFold 3 predictions appear limited by biases in its training data, reflecting the relative scarcity and redundancy of nongenomic nucleic acid structures in the PDB. These findings highlight the potential of AlphaFold 3 for aptamer modeling but underscore the need for further refinement to reliably predict complex, underrepresented structures. AlphaFold 3 represents a powerful step toward in silico aptamer design and offers a promising glimpse into a future where artificial intelligence accelerates discoveries and advancements in aptamers as effective affinity reagents.

## Graphical abstract

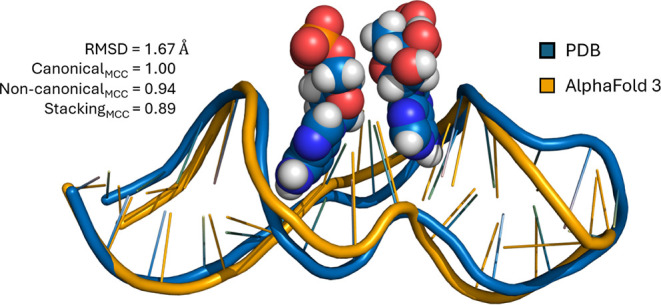

## Introduction

Aptamers are short, single-stranded nucleic acid molecules that have emerged as promising alternatives to traditional affinity reagents, such as antibodies. Their low cost and unique properties—stability, ease of production, and amenability to chemical modification—make them attractive biomacromolecular agents for diverse applications in biotechnology and medicine.^1–4^ Despite these advantages, the process of aptamer discovery remains inefficient. Aptamers are traditionally discovered through an in vitro selection method known as systematic evolution of ligands by exponential enrichment (SELEX), introduced in 1990,^5–7^ whereby large oligonucleotide libraries are screened to select functional aptamer sequences by evolving a random sequence pool using successive rounds of target incubation followed by recovery and polymerase chain reaction (PCR) enrichment of target-bound sequences. While SELEX is deservedly credited with introducing aptamers as alternative ligands, the SELEX process itself is time-consuming and suffers from low success rates and sequence bias due to PCR enrichment of sequences during each screening round.^8,9^

While non-SELEX screening approaches have been reported to address these challenges,^10–20^ aptamer screening platforms operate largely as a “black box” due to the poorly understood relationship between nucleic acid sequence and structure-binding function.^21^ This knowledge gap in aptamer–target systems stands in stark contrast to the detailed understanding of interactions between genomic nucleic acid sequences and their biological protein targets. Thus, the de novo design of aptamer structures remains challenging and further hinders progress and broader acceptance of aptamer technology. Consequently, the aptamer field lags behind commercial antibodies, and the SELEX method, while cumbersome and inefficient, has remained the most common approach for identifying aptamer sequences for the past 35 years.^22^

A significant barrier to aptamer optimization and rational design lies in the scarcity of structural data for functional nucleic acids, which limits understanding of nucleic acid folding.^23^ As of December 2024, the Protein Data Bank (PDB) contained 19 788 nucleic acid structures compared to 224 025 protein structures. Of these nucleic acid structures, 11 529 are DNA and 8259 are RNA structures. Most of the functional nucleic acid structures in the PDB are RNA sequences that originate from similar families, such as ribozymes, rRNA, and tRNA, and are often crystallized as components of larger biological systems.^24^ There are very few functional single-stranded DNA structures in the PDB, since most are double-stranded genomic sequences with helical duplex conformations. This imbalance of data makes it difficult to create generalizable models and fails to capture the full range of structural diversity possible in RNA and DNA molecules, particularly aptamers.^25^ Aptamers, despite their functional promise, remain largely underexplored, with just 117 DNA structures and 232 RNA structures reported in the PDB as of December 2024. Furthermore, many of these are redundant entries, such as 4DII and 4DIH, reflecting repeated analyses of the same sequences under various solution conditions.

While studies of protein–target systems, such as antibody–antigen complexes, often emphasize detailed atomic-level interactions, the vast majority of aptamer publications focus on reporting new aptamer sequences and prioritize binding affinity measurements over structural characterization, leaving a critical knowledge gap in the aptamer field.^26^ This knowledge gap continues even with the most prominent recent protein target, namely SARS-CoV-2. Detailed structural mapping of relevant antibodies^27^ emerged the same year as the pandemic, in contrast to thorough later reviews^28^ of successful aptamers for SARS-CoV-2 that still lack structural verification. Experimental determination of self-folded aptamer structures has faced numerous technical and analytical challenges. High-resolution techniques such as X-ray crystallography, cryoelectron microscopy (cryo-EM), and nuclear magnetic resonance (NMR) spectroscopy often struggle to resolve the flexible, dynamic conformations and allosteric characteristics of aptamers. For example, cryo-EM and X-ray crystallography struggle to resolve poorly ordered regions, such as unpaired nucleotides common in loop segments of hairpins, while NMR suffers from peak broadening and spectral overlap in complex systems. Alternative methods, such as binding assays performed after sequence mutation, nucleotide deletion, selective enzymatic digestion, and circular dichroism (CD), provide indirect insights into broad structural features and potential binding epitopes. However, these indirect methods are inherently low-resolution and require considerable assumptions in modeling atomic coordinates, starting with computational secondary structure predictions discussed next.

Historically, computational tools for predicting aptamer structures have also faced limitations. Traditional secondary structure prediction algorithms, such as mFold,^29^ RNAstructure,^30^ and ViennaRNA,^31^ algorithmically search for stable secondary structures by minimizing free energy using nearest-neighbor energy parameters derived from empirically measured thermodynamic data sets. Other algorithms, such as CentroidFold,^32^ sample suboptimal secondary structures from a Boltzmann distribution of probable self-folded structures.^33,34^ While these algorithms are computationally efficient, they are primarily limited to Watson–Crick base-pairing interactions with allowances for occasional G/T-U wobbles. These algorithms fail to consider noncanonical structures, such as G-quadruplexes, which arise from noncanonical nucleobase interactions (e.g., Hoogsteen base pairs). Furthermore, these algorithms can generate only 2D secondary structure predictions and thus neglect 3D conformations entirely. Previous multistep pipelines for generating 3D structures require one to first sequentially build oligonucleotide secondary structure from the primary sequence using 2D prediction algorithms such as mFold, then construct equivalent 3D models based on the predicted secondary structure with software such as Assemble2.^35^ Since these 3D computational platforms focus on RNA structure generation, the predicted ribose–phosphate backbone often requires manual replacement with a deoxyribose–phosphate backbone using programs such as VMD Molefacture.^36^ In addition to differences in sugar composition and free energy of base pairing, hybridized RNA and DNA duplexes reportedly differ in their helical structures and backbone flexibility.^37^ These methods of oligonucleotide structure prediction are computationally intensive and underscore the need for direct sequence-to-structure prediction approaches.

Another limitation stems from the lack of a universal vocabulary to describe each secondary structure element in the literature. A hairpin is commonly understood to consist of a hybridized stem with a central unhybridized loop; however, other less common secondary structure elements are sometimes labeled inconsistently. For example, two parallel single-stranded segments disrupting a DNA helical track have been described both as an internal (or interior) loop^18,38–40^ and as a bulge.^41,42^ These inconsistencies in nomenclature can complicate structural annotation, particularly in computational modeling and secondary structure prediction, and underscore the importance of standardized terminology when analyzing self-folded DNA structures.

Recent advancements in machine learning have begun to address limitations in structural prediction and thus offer transformative potential in computationally assisted avenues for aptamer development.^12–14^ Additionally, as highlighted in a recent review,^43^ deep learning models such as MXfold2,^44^ UFold,^45^ and SPOT-RNA^46^ have enhanced secondary structure prediction, while 3D prediction models like RoseTTAFoldNA^47^ represent a new era in nucleic acid structure prediction. Building upon the Nobel Prize-winning success for purportedly solving protein folding, AlphaFold 3 by Google DeepMind now extends its capabilities to 3D nucleic acid modeling, enabling the detailed exploration of RNA and DNA structures. This study evaluates the ability of AlphaFold 3 to accurately predict aptamer structures and aptamer–protein complexes by comparing its predictions to experimentally resolved aptamer structures in the PDB and to well-characterized aptamers from the literature, whose structures are not included in the PDB.

## Results and Discussion

### Predictive Performance on Aptamers in the PDB

To provide a comprehensive analysis of PDB aptamer sequences, AlphaFold was used to predict the structures of unbound (apo) aptamers, aptamers bound to protein targets, and aptamers bound to small molecule targets. Initial analysis compared the canonical base pairs, noncanonical base pairs, and base stacking interactions predicted by AlphaFold in nucleic acid aptamer structures against those in experimentally determined structures from the Protein Data Bank (PDB). Base–base interactions were categorized using the Leontis–Westhof nomenclature, in which interacting edges are labeled with W, H, or S to denote the Watson–Crick, Hoogsteen, and sugar edges of nucleobases, respectively, while glycosidic bond orientation is described by *cis* and *trans.* For example, hydrogen bonding between the Watson–Crick edge of one base with the Hoogsteen edge of another with a *trans* orientation would be designated as a W/H *trans* pair. The Leontis–Westhof classification of base–base interactions was extracted using MC-annotate to compute the Matthews correlation coefficient (MCC) values (Table S1) and quantify how accurately AlphaFold reproduced the presence or absence of each interaction type (Figure 1A). Here, the initial analysis demonstrates that AlphaFold handles many unusual motifs reasonably well, as indicated by moderate to high MCC scores in Figure 1A for the majority of non-W/W *cis* interactions. Nonetheless, compared to canonical W/W *cis* interactions, certain specialized edge interactions between bases, such as W/W *trans* and W/S *trans* appear less accurately modeled by AlphaFold. These discrepancies may arise from subtleties of interactions that are not fully captured by the training data set used by AlphaFold. For example, *trans* base pairs often occur in specialized tertiary contexts and can involve additional factors such as cations that stabilize the geometry.^90^ From a practical standpoint, these MCC values highlight both the promise of AlphaFold as a tool for aptamer design and its current limitations. The strong performance for many W/H *trans*, W/S *cis*, and W/S *trans* interactions observed in Figure 1B suggests that AlphaFold is robust at reproducing noncanonical *syn*–*anti* guanosine arrangements commonly found in quadruplexes and tertiary folds. On the other hand, lower MCC scores for purely H/H pairings and certain sugar edge contacts point to underrepresented areas where AlphaFold may benefit from additional training data.

**Figure 1:**
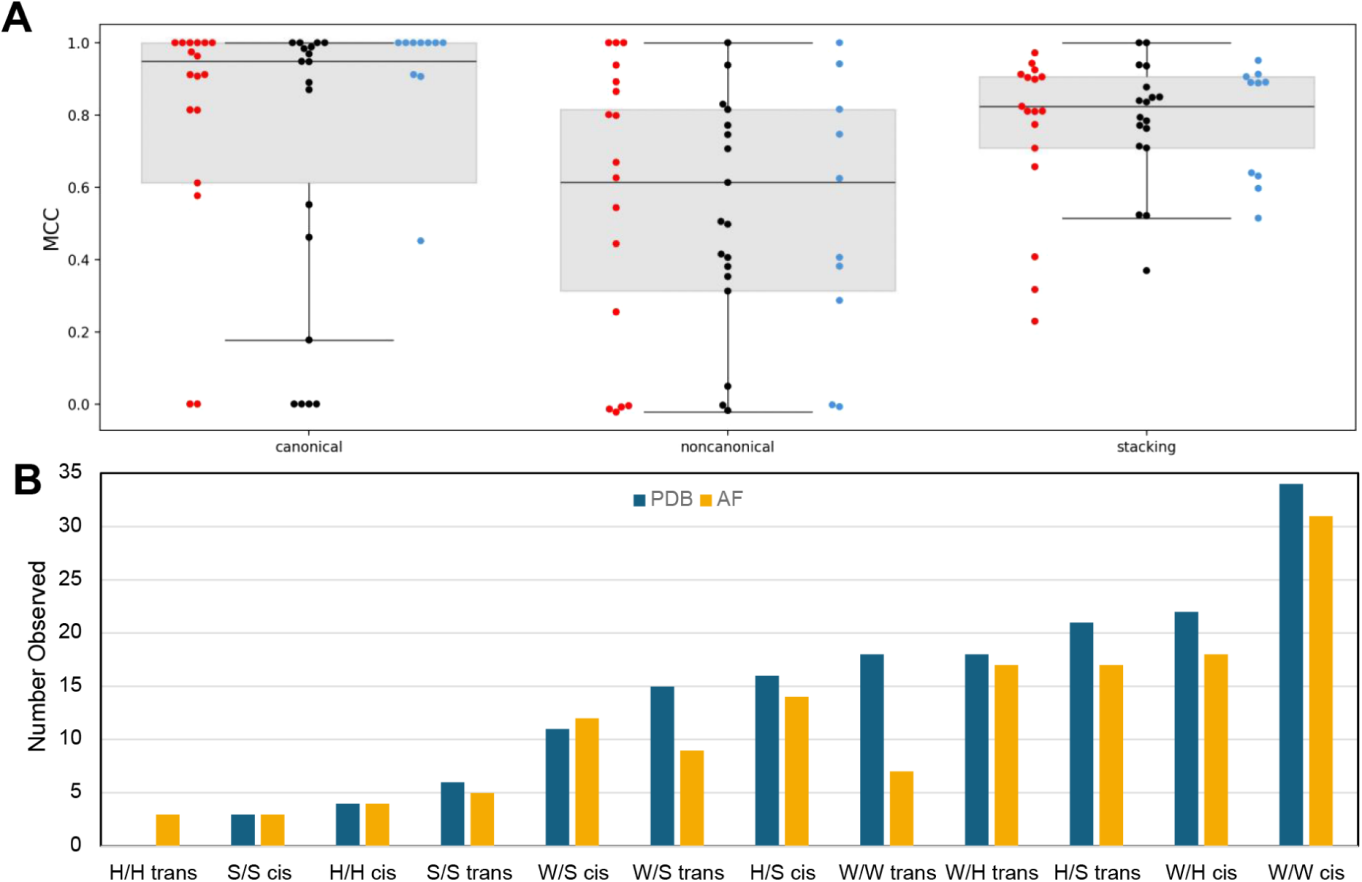
(A) Modified box-and-whisker plots showing the Matthews correlation coefficients (MCC) for canonical, noncanonical, and base stacking interactions predicted by AlphaFold compared to experimentally determined structures in the PDB. The three categories represent apo aptamers (black), aptamer–protein complexes (red), and aptamer–molecule complexes (blue). Each point represents the MCC for a single paired aptamer structure. (B) Bar chart comparing the number of observed base pair interaction subtypes in PDB structures (blue) versus AlphaFold predictions (orange). Base pair interaction subtypes are classified according to the Leontis–Westhof notation, where W, H, and S denote Watson–Crick, Hoogsteen, and sugar edge interactions, respectively.

For each structure prediction, internal AlphaFold confidence metrics were recorded (Table S2) in addition to calculated RMSD values between the predicted and ground truth models for comparison. Table 1 shows a complete list of the PDB structures in this study, as well as selected performance metrics for each aptamer structure. To identify overall data trends, chain pTM values were then plotted as a function of aptamer length (Figure 2A) and RMSD values (Figure 2B). Collectively, these two plots present intriguing patterns that appear independent of the overall structural accuracy of AlphaFold predictions. Figure 2A reveals a positive correlation between aptamer sequence length and predictive confidence, a seemingly counterintuitive result given that combinatorial complexity generally increases with sequence length. As shown in Figure 2B, 80% of the predicted aptamer structures have RMSD values of 2 Å or less, indicating overall strong overlap in predicted 3D structures and structural maps presented in the PDB. Separate analysis using the more localized pLDDT values in Table S2 also shows that 73% of the predicted aptamer structures have RMSD values of 2 Å or less (Figure S1). Together, both pTM and pLDDT values reflect moderate to high confidence scores for at least 65% of aptamer structures predicted by AlphaFold.

**Figure 2:**
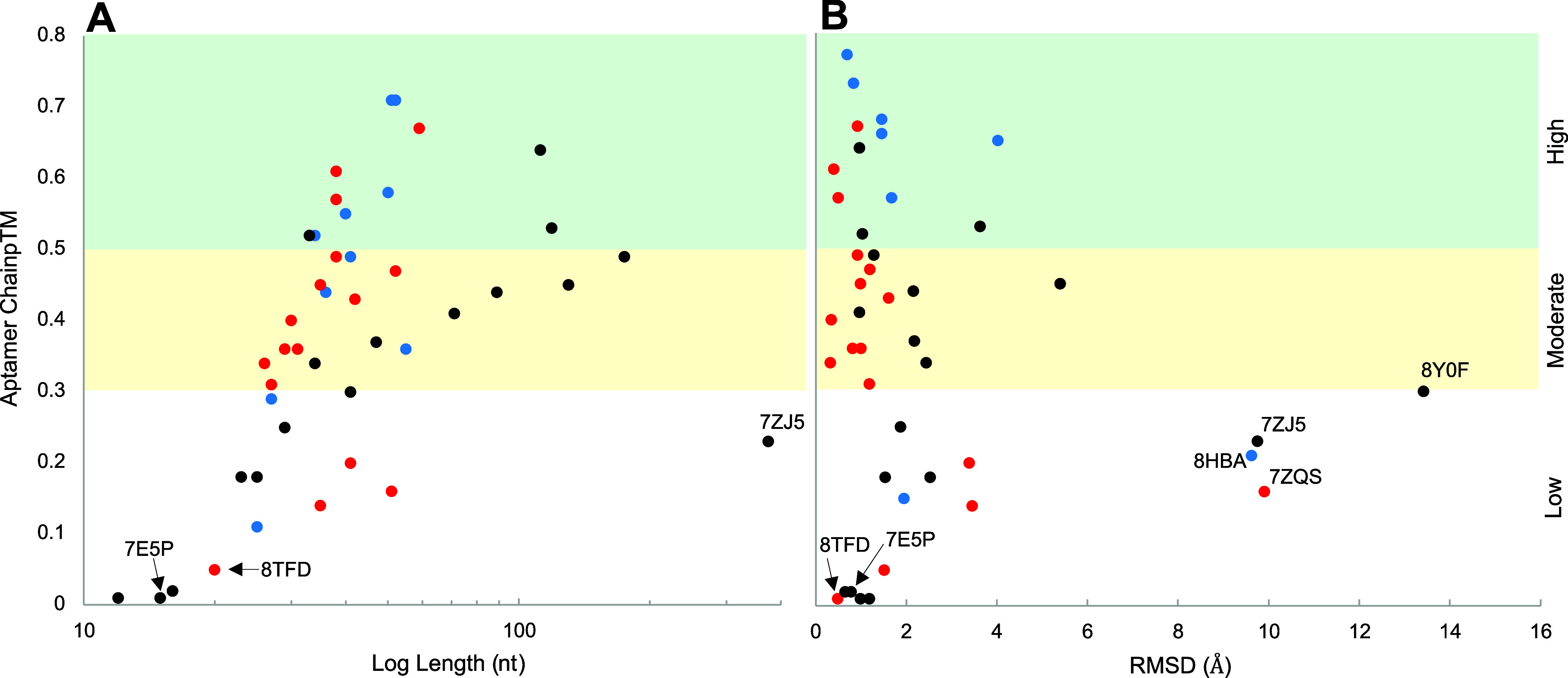
Summary plots of AlphaFold analysis across three aptamer categories: apo (black), aptamer–protein complexes (red), and aptamer–molecule complexes (blue). Aptamer chain pTM scores are plotted as a function of (A) nucleotide length and (B) RMSD values between the AlphaFold predicted and PDB structures. Specific aptamer examples, namely 7E5P and 8TDF, discussed in the main text as well as outliers, are labeled in relevant plots.

**Table 1:**
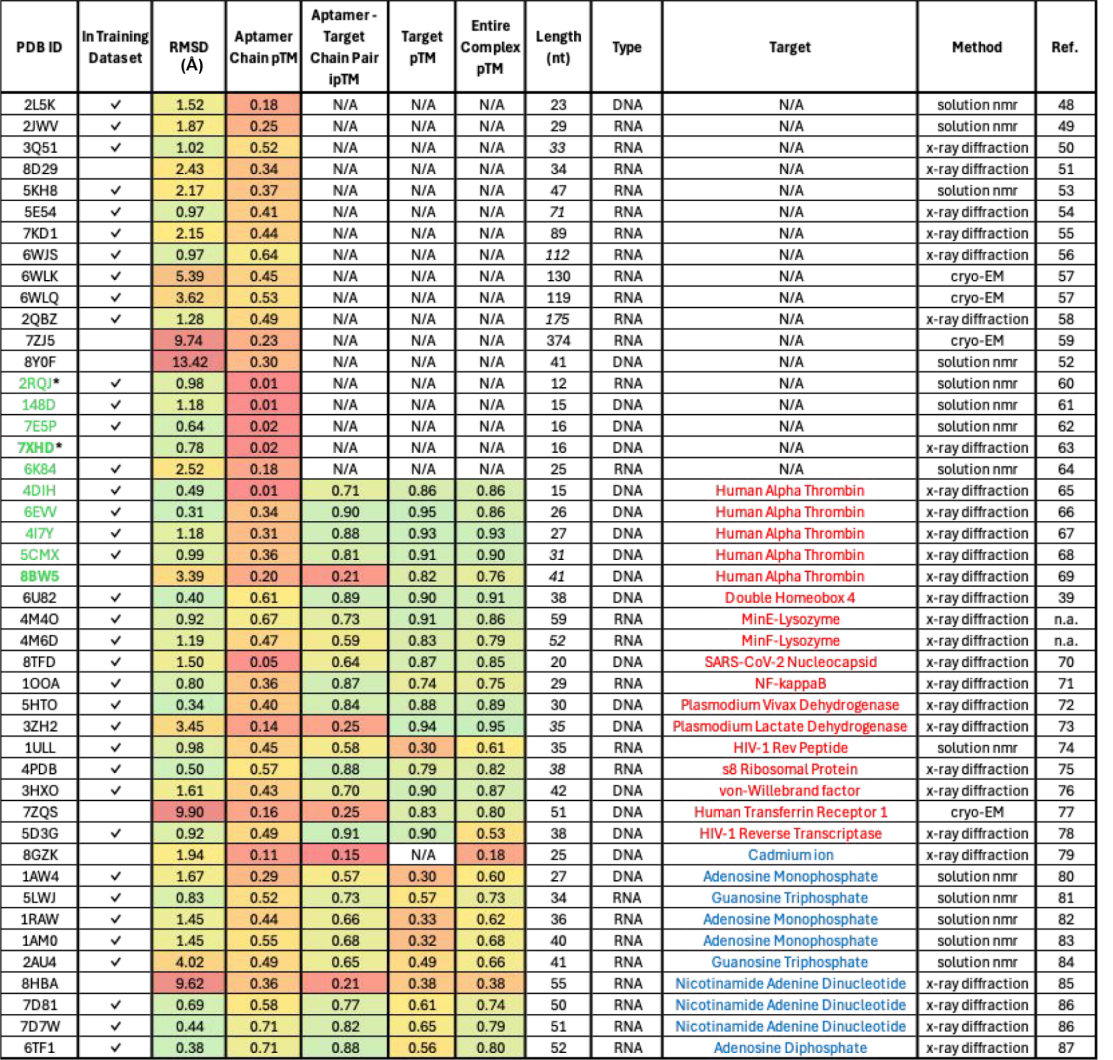
List of the PDB IDs and Key Structural Evaluation Metrics^a^

a PDB IDs associated with G-quadruplex-containing aptamer structures are provided in green text. RMSD values are color-coded from lowest (green, highest structural agreement) to highest (red, lowest structural agreement). Confidence metrics (pTM scores) are similarly color-coded from highest (green) to lowest (red). Italicized numeric entries for nucleotide length indicate PDB structures containing one or more unresolved nucleotides. The text color of a named target indicates aptamer binding to a protein (red) or small molecule (blue). PDB entries uploaded prior to the September 2021 training cutoff date for AlphaFold 3 are indicated with a 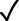 in an adjacent column. Additional data for aptamer homodimers (marked with asterisks) are excluded from this table.

A plausible explanation for the overall trends in chain pTM in Figure 1A,B lies in the composition of the data set used to train AlphaFold, which includes publicly available structures from the PDB in addition to four distilled data sets derived from AlphaFold structures generated using sequences in public repositories. These data sets draw on nucleic acid structures sourced from databases such as Rfam, RNAcentral, and the Nucleotide Collection. Many of the self-folded, natural nucleic acids cataloged in these repositories, such as miRNAs, tRNAs, rRNAs, riboswitches, and ribozymes, span 100 nucleotides or more in length. Consequently, AlphaFold may more readily recognize and reconstruct structures of lengths similar to those in the above databases, thereby increasing its predictive confidence for longer, biologically reminiscent self-folded nucleic acids in comparison to synthetic oligonucleotide sequences bearing abiogenic secondary structure elements.

An additional factor may be the greater inherent flexibility of the aptamer sequences. Synthetic oligonucleotide structures, such as aptamers, often shorter and more conformationally dynamic, are comparatively underrepresented in public databases and are thus limited in representation in the training data set. As shown in Figure 2A, AlphaFold predictions of shorter oligonucleotides routinely elicited low-confidence chain pTM values, despite aligning closely with experimental coordinates. Representative example sequences with 20 nucleotides or fewer, such as those in PDB entries 8TFD^70^ and 7E5P,^62^ produced low chain pTM values yet displayed RMSDs ≤ 1 Å (Figure 2B) and MCC scores ≅ 1 (Table S1) for all interaction types identified, suggesting that low-confidence chain pTM scores do not necessarily correlate with structural accuracy.

All aptamer entries in the PDB up to September 2021 were included in the training set used by AlphaFold 3. A clear effect of training set overlap with AlphaFold 3 becomes apparent by partitioning the data itself according to whether or not an entry met this date cutoff, as indicated in Table 1. AlphaFold achieves an average backbone RMSD of 1.45 Å on structures uploaded before the training cutoff date, in contrast to a 6.40 Å average RMSD for the eight structure entries after September 2021. The same trend is observed by comparing the average chain pTM (0.40 vs 0.22) and interface pTM (0.74 vs 0.41) of structures uploaded before and after the training cutoff date. However, while average performance metric values were reduced for entries uploaded to the PDB after the training cutoff date, half of these eight AlphaFold predictions remained within the 2–3 Å RMSD range and can thus be considered within a range of “high accuracy”, as indicated in Figure 2b. Predicted structures with larger deviations occurred most frequently in aptamers that contain rare, noncanonical elements. Overall, these performance values reflect substantial fidelity to empirical data and underscore the remarkable capacity of AlphaFold to recover native conformations, suggesting a broad applicability to a range of nucleic acid architectures, including the synthetic nucleic acid sequences studied here.

### Comparing AlphaFold Predictions with Prior Analysis of an Historic DNA Aptamer

In 1995, Huizenga and Szostak^91^ reported a 25 nt DNA aptamer that binds adenosine and ATP. Based on site-directed mutagenesis and analog substitution studies, they proposed a model in which the sequence self-folds into a G-quadruplex flanked by hybridized segments, as shown in Figure 3A. Many later studies of this aptamer sequence added stabilizing nucleotides (adenine and thymine) to the 5′ and 3′ ends, respectively. In 1997, follow-up studies by Lin and Patel^80^ proposed a model shown in Figure 3B that shared the same hybridized segments identified by Huizenga and Szostak. However, the NMR spectroscopy studies of Lin and Patel, performed in salt-free solution, elucidated an alternative tertiary fold of the same aptamer when bound to the chemically similar molecule AMP. Notably, they proposed that the AMP-complexed structure (PDB: 1AW4) lacks a G-quadruplex motif due to bound AMP destabilizing G-tetrad formation, as illustrated in Figure 3C. Later in 2024, Edwards et al. revisited the structural features of the ATP–aptamer complex using CD spectroscopy of the aptamer alone and the aptamer complexed with ATP in 100 mM KCl solution. They confirmed the G-quadruplex conformation,^92^ originally proposed by Huizenga and Szostak, who reported using 300 mM NaCl and 5 mM MgCl_2_ solution for SELEX. While only the earliest and most recent studies are highlighted above, the extensive experimental data documenting its binding characteristics and multiple structural states^93,94^ make this historic aptamer an appropriate benchmark for comparative AlphaFold analysis, starting with the apo aptamer and then the target-bound aptamer.

**Figure 3:**
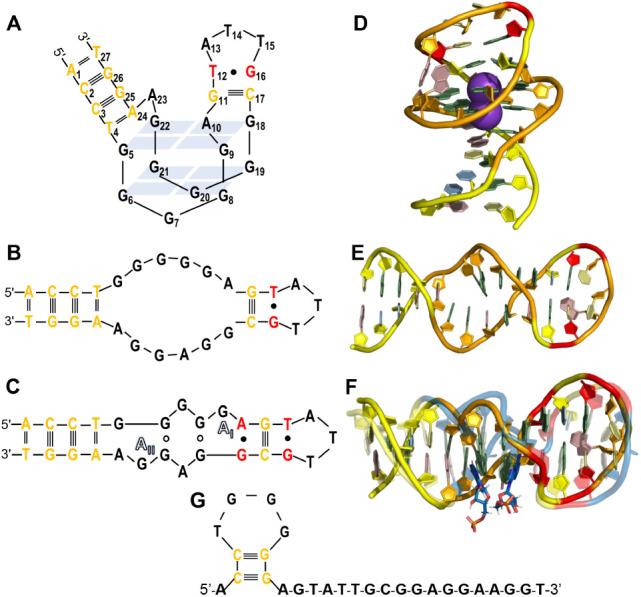
Comparative 2D and 3D structural features of a 27 nt aptamer (PDB: 1AW4) that reportedly binds to ATP and AMP. The 2D secondary structures, each adapted here from cited work for presentation consistency, of (A) the original G-quadruplex forming the structure proposed by Huizenga and Szostak;^91^ (B) proposed conserved base pairs with an internal “bubble”, and (C) the same aptamer bound to two AMP molecules in bold text with Hoogsteen base pairs indicated as open circles (adapted from Lin and Patel^80^). AlphaFold predicted tertiary structures (orange backbone) of (D) the apo aptamer with G-quadruplex in the presence of potassium ions (purple spheres); (E) the apo aptamer in the absence of bound ions; and (F) the aptamer bound to two AMP molecules superimposed with the same complex reported in the PDB (transparent blue backbone), in which central adenine nucleobases are involved in AMP binding. (G) RNAstructure secondary structure prediction of the apo aptamer. (https://rna.urmc.rochester.edu/RNAstructure.html, *accessed December 2024*) Color coding indicates canonical base pairs (yellow) and G•T and G•A wobble pairs (red) in both 2D and 3D structures (A–G) while remaining central bases segments in 3D structures are orange for AlphaFold structures (D–F) and transparent blue for the PDB (F).

When tasked with modeling the apo aptamer in the presence of two potassium ions, AlphaFold successfully predicts a parallel G-quadruplex structure composed of two stacked G-quartets (Figure 3D). This prediction aligns with the original tertiary fold proposed by Huizenga and Szostak and corroborates the CD results by Edwards et al., demonstrating the ability of AlphaFold to recapitulate G-quadruplex structures when informed by appropriate ionic conditions. Notably, in the absence of its molecular target and ions, AlphaFold predicted a strong match to the two hybridized segments but no G-quadruplex structure, as shown in Figure 3E. In the presence of two bound AMP molecules, as shown in Figure 3F, the AlphaFold-predicted structure closely overlaps (RMSD = 1.67 Å) with the superimposed PDB 1AW4 structure, thus agreeing with NMR data from Lin and Patel. In addition to good alignment of the aptamer backbone, AlphaFold faithfully reconstructed nucleobase interactions, perfectly capturing canonical nucleobase interactions (MCC = 1) and most of the noncanonical (MCC = 0.94) and stacking interactions (MCC = 0.89) (Table S1). In contrast to the earliest theoretical and NMR-based models, as well as the AlphaFold predictions, traditional 2D prediction algorithms such as RNAstructure, shown in Figure 3G, failed to capture Hoogsteen base pairs, the G-quadruplex, and even the conserved hybridized segments of the aptamer.

In the context of this historic aptamer, these findings demonstrate that AlphaFold possesses a robust capability to predict diverse aptamer conformations, including G-quadruplexes, and imply its potential as a practical tool for predicting aptamer structures. Earlier one-dimensional computational tools, such as QGRS Mapper^95^ for G-quadruplex prediction, typically ignore Watson–Crick base pairing between nucleotides, whereas AlphaFold captures both canonical and noncanonical base interactions and the resulting secondary structure elements. Thus, starting with other aptamers included in the PDB, the next sections address the attributes and limitations of AlphaFold as a practical tool for predicting general aptamer conformations, as well as specific structural elements.

### G-Quadruplex Structures: A Highlight

Here, the analysis was extended to ten aptamer sequences with reported G-quadruplexes, as shown in the green text in Table 1. The discrepancy between AlphaFold confidence metrics and structural accuracy was most evident in these G-quadruplex conformations, which comprised many of the shortest oligonucleotide structures surveyed in this study. Despite low confidence metrics, AlphaFold accurately captured both parallel and antiparallel quadruplexes, as illustrated in Figure 4A,B, respectively. Noncanonical and stacking MCC values for G-quadruplex-containing aptamers were among the highest across the data set (Table S1), often exceeding 0.9. This analysis further supports previous observations that AlphaFold successfully reconstructs the stacked guanine tetrads that define the G-quadruplex structures. It even succeeded in predicting more exotic quadruplexes, exemplified by PDB entry 4I7Y in Figure 4C, in which a duplex and a highly distorted G-quartet stack form a “pseudoquadruplex”.^67^ Even in this atypical arrangement, AlphaFold achieved reasonable structural alignment (RMSD = 1.18 Å), perfectly paired every Watson–Crick base interaction in the duplex, and most of the noncanonical base interactions in the pseudoquadruplex (MCC = 0.8). The unusual stacking conformation of the pseudoquadruplex domain in the AlphaFold prediction was also impressively accurate, with a nearly perfect base-stacking MCC score of 0.97.

**Figure 4:**
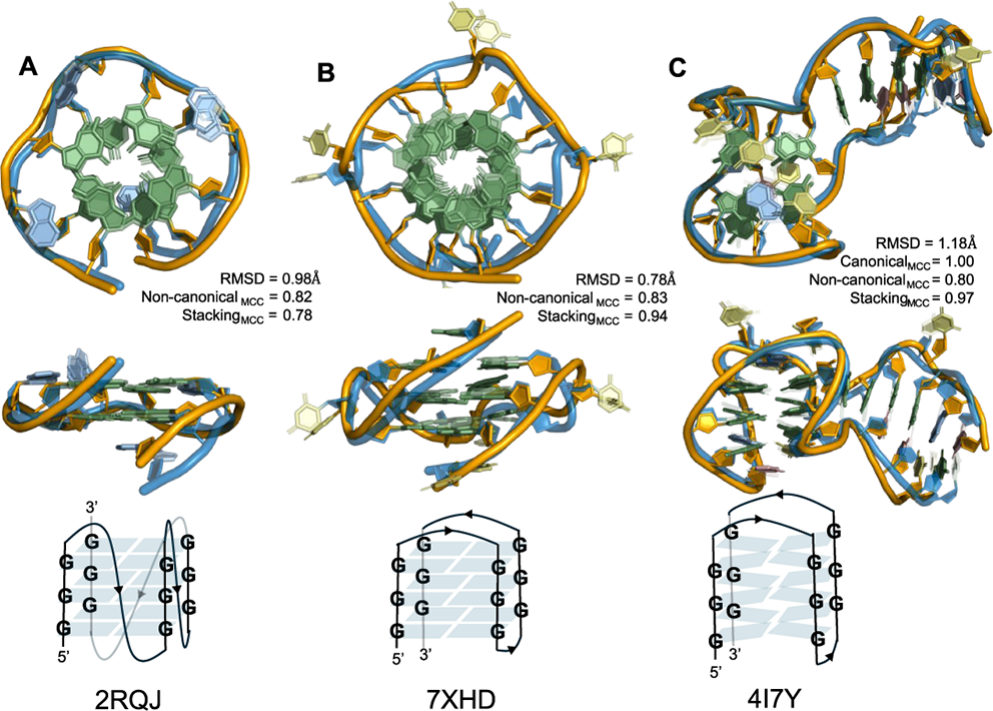
AlphaFold predictions of aptamers (orange backbone) superimposed over PDB structures (blue backbone), forming three distinct types of G-quadruplex structures. Each aptamer is shown in top (*top row*) and side (*middle row*) views: (A) an RNA aptamer adopting a parallel G-quadruplex; (B) a DNA aptamer forming an antiparallel G-quadruplex; and (C) a DNA aptamer adopting an antiparallel pseudoquadruplex conformation. Schematic representations of their quadruplex folding patterns and corresponding PDB IDs are provided below each structure.

The consistent convergence with experimentally resolved G-quadruplex coordinates and high performance in predicting *syn*–*anti* guanosine base interaction types suggest that AlphaFold’s success may stem from the substantial representation of these secondary structure elements in its training data. G-quadruplexes are prevalent and biologically relevant nucleic acid structures commonly found in telomeres and gene promoter regions in genomic DNA and in untranslated regions of RNA. Their relevance as structural motifs in processes such as genome stabilization, transcriptional regulation, and telomere maintenance has driven extensive experimental research, resulting in a wealth of high-resolution data for G-quadruplex structures across a variety of conformations.

The abundance of data in the PDB containing natural G-quartet structures likely contributes to the remarkable ability of AlphaFold to recognize and accurately reconstruct G-quadruplexes, even in cases where its internal confidence metrics suggest lower certainty. The decorrelation between RMSD and pTM values suggests that AlphaFold can achieve structural fidelity even when its confidence metrics are not strongly supportive. These findings highlight its learned ability to resolve stable global folds and accurately capture essential architectural features of nucleic acid structures. Notably, in contrast to other models, AlphaFold is the first known computational tool capable of accurately predicting 3D G-quadruplex nucleic acid structures directly from primary sequences.

### Role of Intermolecular Interactions

Intermolecular interactions appear to enhance the AlphaFold confidence scores when predicting oligonucleotide structures, as illustrated by the inclusion of ionic cofactors. Beyond adjustments to confidence scores or structure accuracy, including ionic cofactors is sometimes necessary for AlphaFold to predict key structural features. For example, PDB entry 8BW5 is the X-ray structure of the *M08s-1* aptamer complexed with thrombin.^69^ It features both duplex and antiparallel G-quadruplex domains connected through a purine-rich junction that results in a unique orientation in which the G-quadruplex central axis is roughly orthogonal to the duplex axis. AlphaFold correctly folds the duplex domain but fails to predict the G-quadruplex of *M08s-1* complexed with its protein target unless a sodium ion is present in the assembly. Including a sodium ion enables AlphaFold to fold the G-quadruplex domain with the correct antiparallel conformation and an improved MCC score for base-stacking interactions, but with relatively weak structural alignment to the ground truth aptamer structure in the PDB (Figure 5). Furthermore, AlphaFold misidentifies the protein-binding epitope, predicting interaction with the C-terminus instead of the N-terminus (Figure S2).

**Figure 5:**
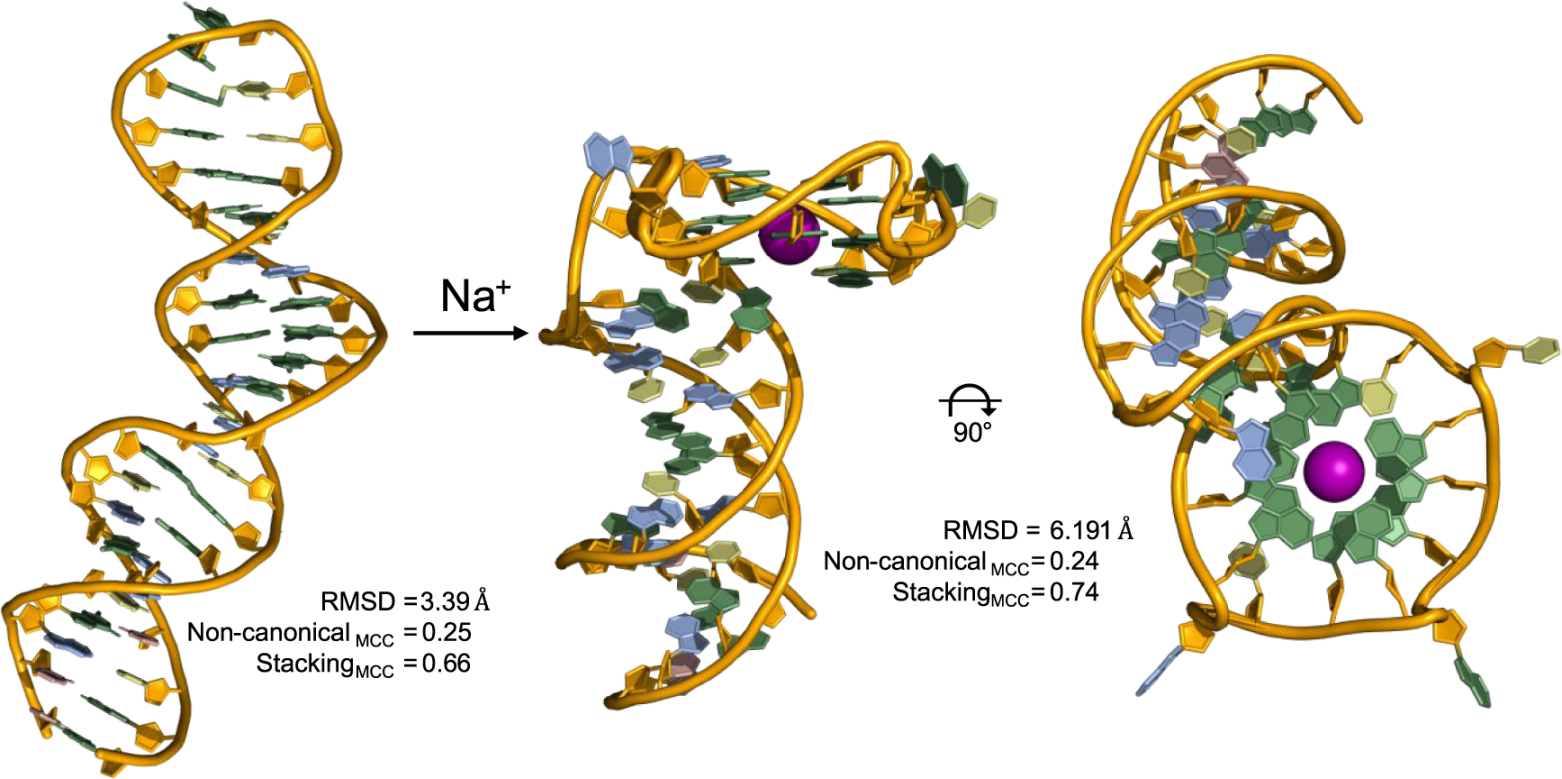
Effect of adding Na^+^ (represented by purple sphere in the middle and right panels) on AlphaFold prediction for the *M08s-1* DNA aptamer structure from PDB ID: 8BW5. Bound thrombin targets are not shown for simplicity.

The inclusion of ionic cofactors did not improve the accuracy of the AlphaFold predictions in all cases. For example, a rotational error was observed when predicting the thrombin-binding DNA aptamer *HD1*, represented by PDB entry 4DIH. *HD1* adopts an antiparallel G-quadruplex stabilized by sodium ions^65^ and features a palindromic sequence comprised of dG-dT repeats. Incorporating a sodium ion into the AlphaFold prediction slightly enhances structural fidelity (ΔRMSD = −0.06 Å) and confidence ( Δ chain pTM = +0.02). However, while AlphaFold still predicts an antiparallel G-quadruplex topology with the addition of sodium ions, reflecting the PDB structure, it erroneously rotated the quadruplex around its central axis. Rotational errors are consistent with known challenges in resolving structures exhibiting rotational symmetry by AlphaFold. While predicting atomic coordinates without enforcing global symmetry constraints simplifies its design, this approach can introduce ambiguity when determining structures with rotational symmetry or repetitive secondary structure elements such as G-quadruplexes and palindromic sequences exemplified by the *HD1* aptamer. The stochastic nature of the diffusion model driving AlphaFold predictions can thus favor one of several equivalent orientations, leading to misalignments between the aptamer and the target binding domain.^96^ Despite these issues, the ability to approximate functional interactions highlights the modeling strengths of AlphaFold, even when dealing with symmetric assemblies.

In contrast to the *M08s-1* aptamer discussed earlier, intermolecular interactions with bound proteins often markedly enhance the accuracy of AlphaFold-predicted aptamer structures. This improvement can be demonstrated by revisiting PDB entry 4DIH, the *HD1* aptamer complexed with thrombin, and comparing it to PDB entry 148D, which features the same *HD1* aptamer in its apo state.^61^ AlphaFold models the *HD1* aptamer chain in its apo state with an RMSD of 1.18 Å but more accurately predicts the aptamer structure in its bound state with thrombin, yielding a more aligned backbone RMSD of 0.49 Å and demonstrating how AlphaFold leverages stabilizing interactions with proteins to more confidently model aptamer structures. Across all surveyed aptamers, protein-bound aptamer structures were consistently the most accurately and confidently predicted subset. AlphaFold achieved an average RMSD of 1.78 Å for protein-bound aptamer chains, outperforming both small molecule-bound aptamers (average RMSD = 2.25 Å) and apo aptamers (average RMSD = 2.92 Å). This disparity reflects its ability to aptly resolve the intricate surface topologies of protein scaffolds, which often provide more extensive interaction networks than small molecules.

These findings align with the design principles of AlphaFold, which prioritize accurate prediction of protein structures. Protein scaffolds may act as stabilizing frameworks that guide aptamer folding by reducing conformational entropy, effectively narrowing the solution space explored by its diffusion model. The presence of protein interactions likely introduces additional physical constraints, anchoring aptamers within locally stable structural conformations. The proficiency of AlphaFold’s protein structural prediction plays a key role in this improvement, as the model is optimized for solving protein folding and interfaces, as described in the foundational architectures of earlier AlphaFold versions and the most recent release.^96,97^

### Challenges with Rare and Synthetic Secondary Structure Elements

The limitations of AlphaFold become evident when predicting structures with fewer intermolecular interactions or rare synthetic motifs. These challenges are particularly apparent in apo aptamer structures and aptamers bound to small molecular targets, which often feature flexible, looped segments and secondary structure elements arising from noncanonical base interactions critical for their binding function. Such features, which lack the stabilizing constraints of protein or larger molecular scaffolds, often lie outside the most confident predictive range of the model. A notable example is PDB entry 8HBA, the X-ray structure of an NAD-II riboswitch aptamer domain bound to two NAD molecules.^85^ As shown in Figure 6, this structure features several complex and even noncanonical secondary structure elements, including a long-range pseudoknot, a triple helix formed between a five-adenine strand and a duplex, and two distinct quadruple-base interactions that stabilize the core of the riboswitch. AlphaFold accurately predicted structural domains stabilized by canonical nucleobase interactions (MCC_canonical_ = 0.88) associated with the duplexes. However, it struggled to accurately model the intricate pair of quadruple–base interactions at the riboswitch core, which are stabilized by stacking (MCC_stacking_ = 0.47) and various noncanonical edge interactions such as W/W *trans* and W/S *trans*, as reflected by a low MCC score of −0.01 for noncanonical interactions. These unresolved elements significantly affected the overall alignment with the experimental structure, yielding a misaligned RMSD of 9.62 Å, as shown in Table 1. In contrast to several G-quadruplex-forming aptamers highlighted previously, the inability to fully capture the riboswitch core reflects the challenges posed by multiple neighboring secondary structure elements arising from noncanonical base interactions, which require precise modeling of intricate inter-residue networks and long-distance structural dependencies.

**Figure 6:**
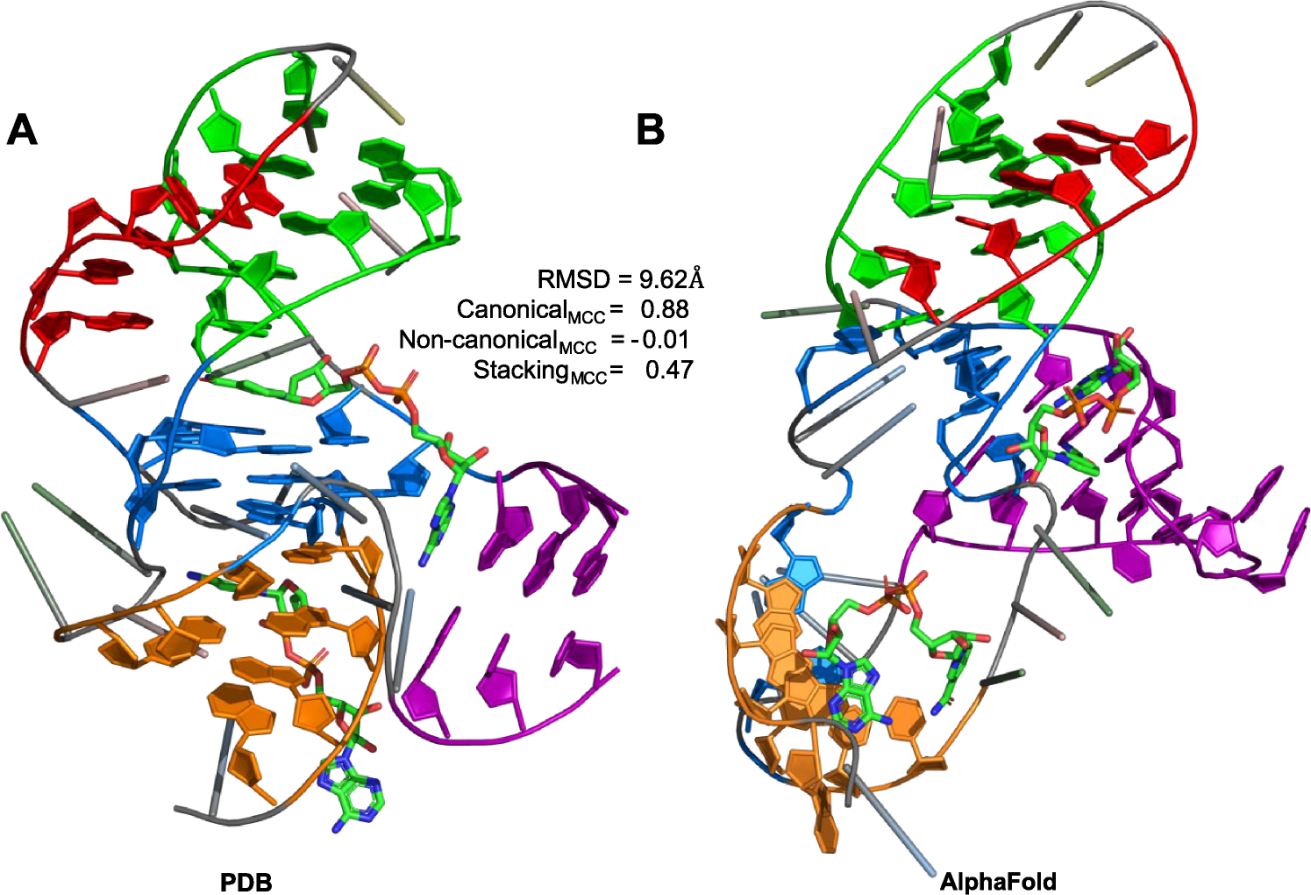
Structural comparison of the (A) experimentally determined PDB structure and (B) AlphaFold-predicted model for the riboswitch (PDB ID: 8HBA) bound to two NAD molecules. Key structural elements identified in the PDB structure are depicted by ring structure representations and color-coded as follows: quadruple base interactions (blue), pseudoknot (orange), duplex (purple), and triplex (green) with the five-adenine segment (red).

Similarly, AlphaFold struggled with the apo DNA aptamer *Sgc8c* from PDB entry 8Y0F, an aptamer structure determined through solution NMR featuring a unique three-way junction stabilized by nucleobase-backbone and nucleobase stacking interactions.^52^ These intricate tertiary interactions presented significant modeling challenges, as demonstrated by poor MCC scores for both canonical (0.17) and noncanonical (0.42) interactions (Table S1). While the inclusion of Mg^2+^ modestly improved its alignment, enabling it to capture the overall aptamer topology, AlphaFold still failed to resolve the three-way junction, resulting in the highest RMSD (13.42 Å) of all the aptamer cases listed in Table 1.

PDB entry 7ZJ5, a synthetic RNA origami construct designed to facilitate high-resolution cryo-EM imaging of two conjugated aptamer segments, further highlights limitations of AlphaFold.^59^ This 374-nucleotide-long scaffold includes fluorescent *pepper* and *broccoli* aptamer domains, each presenting distinct structural challenges, as illustrated in Figure 7. Similar to PDB ID 8Y0F discussed above, AlphaFold failed to predict the three-way junction motif within the *pepper* aptamer domain but did successfully resolve a G-quadruplex in the neighboring *broccoli* domain. This contrast underscores its greater proficiency with well-studied secondary structure elements, such as G-quadruplexes, which are more prevalent in its training data. Despite its difficulties with backbone alignment, AlphaFold remarkably captured most canonical base pairs (MCC = 0.98) and stacking interactions (MCC = 0.84) (Table S1) of this large synthetic construct, demonstrating its utility in providing coarse structural approximations.

**Figure 7:**
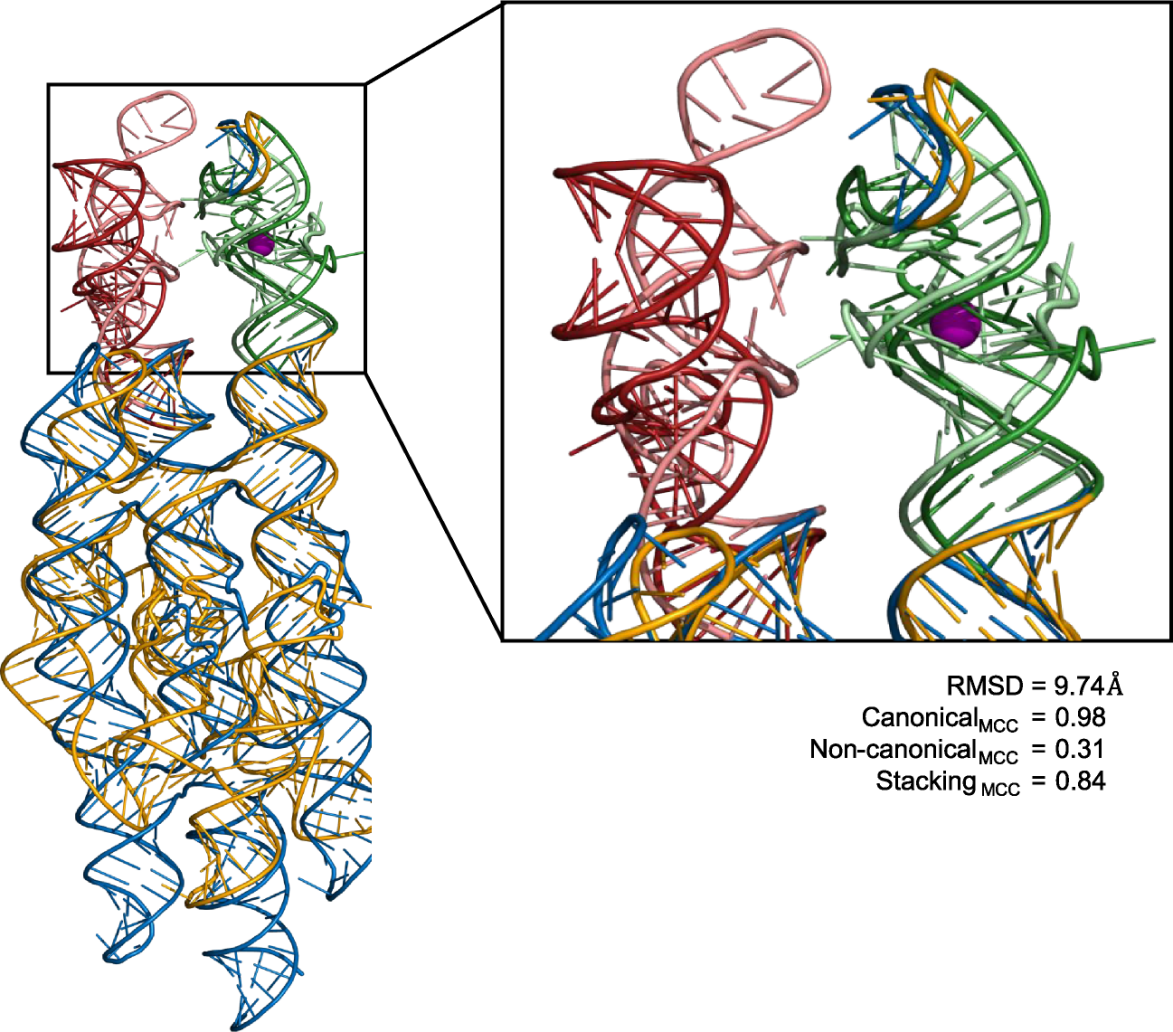
PDB ID: 7ZJ5 with its RNA origami scaffold (blue), *pepper* aptamer domain (light red), and *broccoli* aptamer domain (light green), superimposed over AlphaFold prediction for the scaffold (orange), *pepper* domain (dark red), and broccoli aptamer domain (dark green). Associated potassium ion is shown as a purple sphere.

It is worth noting that experimental methods themselves can introduce variability into ″ground truth” structures. Obtaining an atomic-level resolution is often challenging. Many aptamer structures have been solved by using techniques such as cryo-EM and solution NMR, which likely contribute to the degree of accuracy of AlphaFold predictions. Structural heterogeneity and the flexible or dynamic nature of aptamer structures can negatively affect resolution in solution NMR. Cryo-EM-derived structures, such as PDB entries 7ZQS,^77^ 6WLK,^57^ and 6WLQ,^57^ with an average RMSD of 6.30 Å, often require additional inference to resolve local atomic positions, especially for diffuse or flexible regions, which can lead to noisier experimental coordinates. These challenges likely contribute to the relatively poor alignment observed in such structures compared to other data sets, pointing out an inherent difficulty in benchmarking the performance of AlphaFold against experimental data where structural uncertainty exists.

### Predicting Structurally Uncharacterized Aptamers and Non-PDB Aptamers

While several specific exceptions have been detailed above, AlphaFold has overall demonstrated exceptional proficiency in matching its predictions to multiple known aptamer structures listed in Table 1. Its true transformative potential, however, lies in its ability to accurately model the 3D structures of aptamer sequences that are not yet structurally solved and, in turn, also uncover their potential interactions with molecular targets. To further explore its capabilities, AlphaFold was challenged with predicting non-PDB aptamers lacking experimentally solved tertiary structures, but whose binding characteristics, beyond binding affinity measurements, have been extensively studied in the literature.

Roychowdhury-Saha et al. identified a family of FAD-binding sequences through SELEX, including an aptamer named *Ftest1*, which featured a conserved 5′-CAAGAA-3′ motif critical for binding.^98^ AlphaFold modeled the *Ftest1*-FAD complex, shown in Figure 8, with moderate yet significant confidence, assigning it a complex pTM score of 0.56. Notably, the conserved motif was localized near the isoalloxazine moiety of FAD and appeared to play an important role in binding with the three-ring structure. This mechanism of interaction, where aptamers bind to the isoalloxazine moiety of flavin molecules, has been demonstrated before in PDB entries 7RWR,^99^ 2YIE,^100^ and 3F2Q.^101^ Similarly, Wang et al. characterized a separate FAD-binding aptamer, *FAD-5s*, for which AlphaFold predicts the *FAD-5s*–FAD complex with a chain pair ipTM of 0.62, suggesting confidence in the interaction. This prediction aligns with experimental data, supporting the capacity of the aptamer for ligand binding.^102^

**Figure 8:**
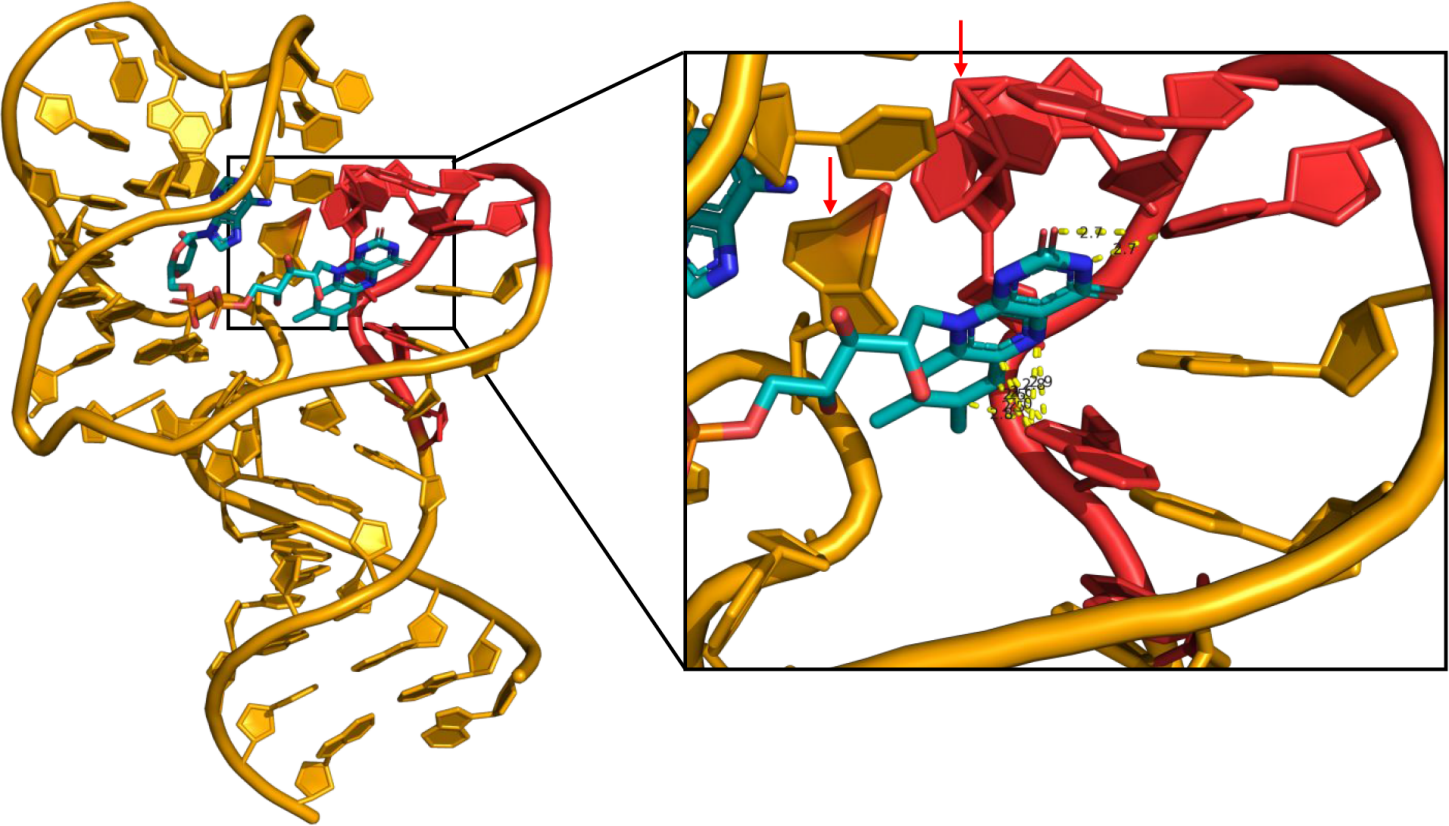
AlphaFold prediction of the non-PDB aptamer called *Ftest1* bound to FAD. The conserved nucleotide motif (5′-CAAGAA-3′) shown in red, identified in several sequence families after the final round of SELEX, is localized near the isoalloxazine moiety. Unrealistic atomic artifacts produced by AlphaFold are highlighted with red arrows.

In certain instances, particularly with DNA structures, AlphaFold generates artifacts where nucleotides appear unrealistically fused or morphed together, as highlighted with red arrows in the yellow conserved motif of *Ftest1* in Figure 8. These anomalies may result from training performed predominantly on protein data, leading to less precise representations and occasional artifacts in DNA geometries and synthetic sequences that do not match well with evolutionary motifs. This observation underscores the necessity for cautious interpretation of nucleic acid predictions by AlphaFold and suggests a need for further refinement in modeling DNA structures.

Additional validation of AlphaFold and its ability to model non-PDB aptamer–target interactions comes from its structural predictions of two streptavidin aptamers. Bing et al. observed that streptavidin-binding aptamers identified by independent laboratories shared a conserved loop, suggesting a common mechanism of interaction with streptavidin.^38^ Two of these aptamers, *St-2–1* (discovered by Bing et al.) and *StreptApt5* (discovered by Ruigrok et al.)^103^ were selected for analysis with AlphaFold. Consistent with mFold predictions, AlphaFold modeled the apo structures of these aptamers (Figure S3), producing intrastrand base pairing patterns that aligned with secondary structures reported by both Bing et al. and Ruigrok et al.

Both experimental studies demonstrated that biotin competes with *St-2–1* and *StreptApt5* for binding to streptavidin, suggesting that these aptamers bind to the same epitope as biotin. Interestingly, when tasked with modeling the aptamer–streptavidin complexes, AlphaFold localized nucleotides from both aptamers deep within the biotin-binding pockets on the protein surface, as shown in Figure 9. This result further demonstrates that AlphaFold can place aptamers within plausible target-binding epitopes, consistent with experimental findings. However, in contrast to the self-folded apo aptamer structures (Figure S3), AlphaFold modeled the target-bound aptamers as unfolded chains wrapped around the streptavidin tetramer, seated within the interfacial groove formed between two streptavidin dimers, eliminating key secondary structure elements such as hairpins, bulges, and duplexes predicted in the apo structures. Additionally, the nucleotides positioned within the binding pockets differed from those identified as critical for binding by Ruigrok et al., raising the possibility of alternative binding modes or intermediate structural states. This discrepancy may hint at potential allosteric behavior in these aptamers.

**Figure 9:**
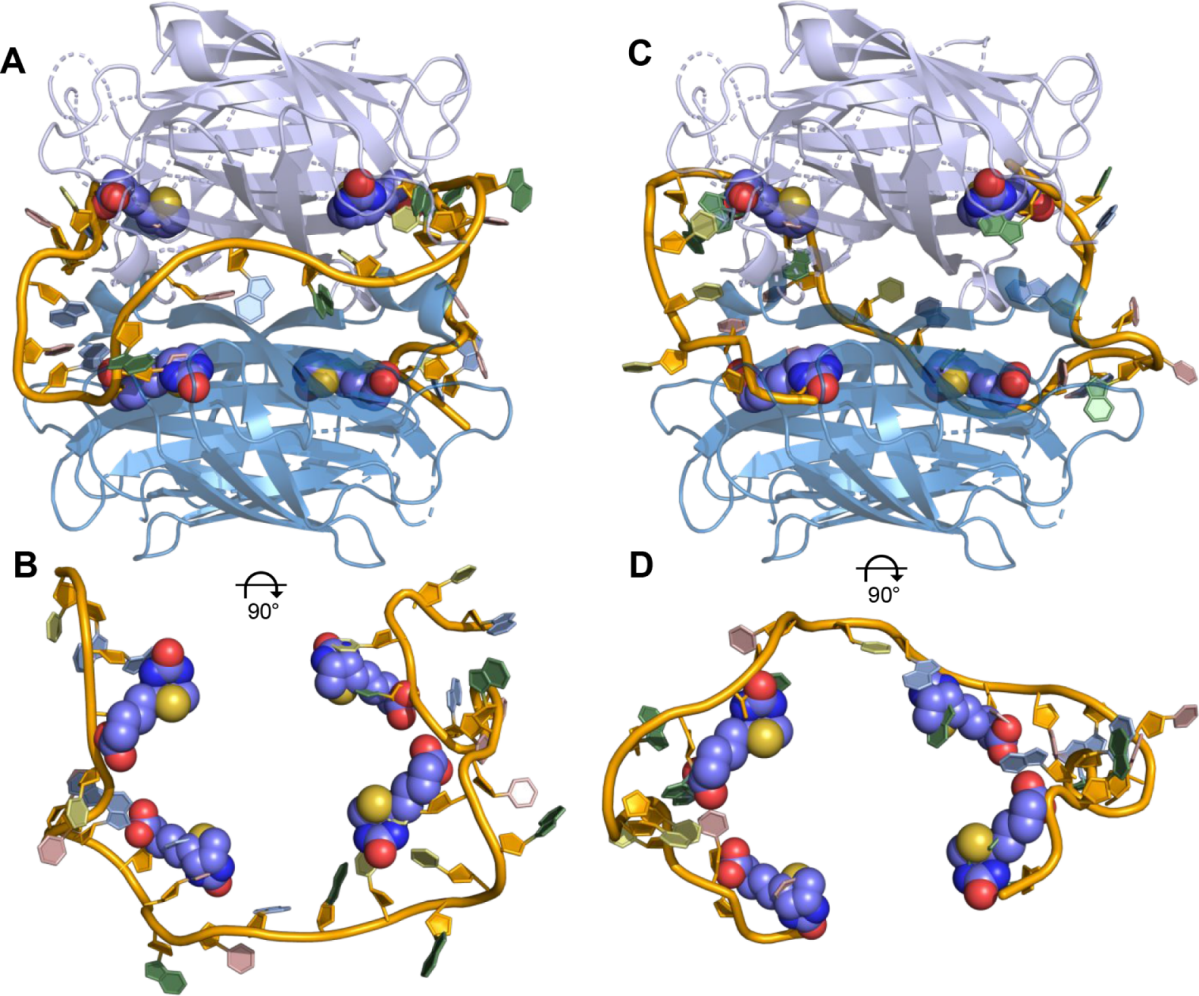
AlphaFold predictions of non-PDB streptavidin-binding aptamers, showing the aptamer (orange backbone) bound to tetrameric streptavidin (light purple and blue). The top panels display the full aptamer–streptavidin complexes, while the bottom panels show the same aptamer structures alone to highlight their bound conformations. (A, B) The *St-2–1* aptamer and (C, D) the *StreptApt5* aptamer are depicted to interact with four biotin molecules (represented with a space-filling CPK model) within the streptavidin-binding pockets.

### Non-PDB Aptamers with Experimentally Verified G-Quadruplexes

Next, two reported aptamer sequences from the literature were selected, which have been shown to form parallel G-quadruplexes through CD measurements. The first sequence, *S14,* a 44 nt DNA aptamer against the spike trimer antigen of SARS-CoV-2 identified by Gupta et al., was predicted by QGRS Mapper to fold into multiple overlapping G-quadruplex motifs with a G-score of 71.^104^ Gupta et al. verified quadruplex formation by measuring a negative peak at ∼240 nm and a positive peak at ∼260 nm within the CD spectrum of *S14*, indicating a parallel G-quadruplex conformation. Remarkably, in agreement with the CD measurements of Gupta et al., AlphaFold reproduced this parallel G-quadruplex when *S14* was modeled in the presence of three potassium ions, reflecting the 50 mM KCl conditions used by Gupta et al. (Figure S4) during experimental characterization. AlphaFold models the G-quadruplex aptamer with a chain pTM score of 0.34, indicating moderate confidence, and suggests that the predicted structure, while consistent with experimental findings, should be used with discretion. Intriguingly, when potassium ions were omitted from the modeling environment, AlphaFold consistently folded *S14* into a hybrid G-quadruplex conformation, underscoring the importance of simulating relevant ion conditions to achieve accurate structural predictions.

As a second test case, a bispecific aptamer against VEGFR-1 and VEGFR-2, named *Apt02*, identified through SELEX by Yoshitomi et al., was selected for analysis with AlphaFold.^105^ Yoshitomi et al. reported that the QGRS mapper predicts that *Apt02* is likely to form a G-quadruplex with a G-score of 20. Subsequent CD measurements showed a negative peak at ∼240 nm and a positive peak at ∼260 nm, suggesting *Apt02* folds into a parallel G-quadruplex conformation. Consistent with these findings, AlphaFold also predicts a parallel G-quadruplex conformation for *Apt02*, with a moderate aptamer chain pTM of 0.36 (Figure S5). These results further demonstrate that AlphaFold can accurately model G-quadruplex structures, particularly in cases concerning non-PDB aptamer sequences.

The performance of AlphaFold with non-PDB aptamers underscores both its strengths and its limitations. It excels at localizing key binding epitopes and modeling general interaction patterns, but its accuracy can decline for complex or noncanonical secondary structure motifs, particularly for novel motifs lacking well-represented experimentally resolved 3D coordinates. Nonetheless, the ability of AlphaFold to propose structural hypotheses for uncharacterized aptamer–target interactions renders it a powerful tool for advancing aptamer design and uncovering principles of nucleic acid–target recognition, especially in scenarios where experimental data are limited or difficult to obtain.

## Conclusions

Employing AlphaFold for aptamer structure prediction has revealed remarkable strengths alongside key limitations, providing a nuanced understanding of its capabilities. Across a broad spectrum of aptamers with and without bound targets, including both PDB-derived and non-PDB sequences, AlphaFold has demonstrated exceptional accuracy in predicting 3D structural coordinates, often achieving a backbone RMSD ≤ 2 Å. A critical strength of AlphaFold lies in its ability to model diverse aptamer architectures, ranging from small, structured motifs to complex, protein-bound assemblies. However, systematic analyses reveal that the confidence metrics do not always correlate with structural accuracy. For example, while short sequences exhibited low chain pTM scores, their predicted structures aligned closely with experimental data, often achieving RMSD values of less than 1 Å. In contrast, while many G-quadruplexes were accurately predicted, AlphaFold was less successful in predicting other noncanonical secondary structure elements, such as pseudoknots and long-range nucleobase interactions. Furthermore, lower accuracy on structures uploaded to the PDB after the training cutoff date provides supporting evidence that the current AlphaFold 3 model performs well overall if there is sufficient exposure to specific structural elements during training but becomes less reliable for modeling novel or infrequent structural motifs. Specific examples of newer PDB entries with rare structures that are likely to fill gaps in the training set include 824N (i-motif), 8FI2 (4-way junction), and 7SZU (base quadruple•base-triple stack). These challenges underscore the influence of the data set composition used to train AlphaFold, which is heavily biased toward canonical, biologically evolved nucleic acid motifs.

Yet, its impressive performance with well-represented noncanonical secondary structure elements, such as G-quadruplexes, showcases its strengths as a predictive tool. Until now, no computational tool existed that could accurately generate 3D structures of G-quadruplexes from the primary structure. AlphaFold predictions successfully capture many of the noncanonical nucleobase interactions that underpin quadruplex motifs, including W/H *cis* base-pairing and tetrad stacking, marking a milestone in nucleic acid research that sets it apart from traditional tools such as QGRS Mapper, RNAstructure, and mFold, which are limited to one- and two-dimensional predictions. Additionally, AlphaFold appears to identify potential binding pockets and interaction epitopes within protein surfaces, implying that it has learned general principles of molecular recognition, especially in the case of proteins. Overall, the predictions offered by AlphaFold should be viewed as data-informed yet speculative. They can provide valuable starting points for experimental exploration and serve as guides for designing mutagenesis studies, binding assays, and structural determination efforts. By accurately localizing aptamers within plausible binding regions, we provide a powerful framework for probing nucleic acid–protein interfaces. By integrating AlphaFold predictions with experimental approaches, researchers can streamline current screening and characterization approaches for synthetic nucleic acids. As the quantity of structural data increases and additional novel secondary structure elements are solved, the predictive power of AlphaFold will likely improve as future versions are trained on more robust data sets that capture underrepresented features of structural conformation space. Thus, this latest AlphaFold release marks a critical step toward the rational design of aptamers de novo and in silico aptamer selection, providing a powerful supplemental platform for advancing the discovery of molecular affinity reagents.

## Methods

### Aptamer Data Set

A total of 45 nucleic acid aptamer structures were retrieved from the Protein Data Bank (PDB; accessed December 2024) to represent a diverse series of aptamer conformations, targets, and sequence lengths. Italicized lettering is used in the main text to distinguish the occasional use of the original aptamer name from its PDB ID (e.g., *M08s-1* vs PDB ID 8BW5 to designate the same aptamer sequence). This data set is divided into three aptamer categories: (i) 18 apo aptamer structures^48–64^ in the absence of a bound target, ranging from 12 to 374 nucleotides (nt) in length; (ii) 17 aptamer–protein complexes,^39,65–78^ ranging from 15 to 65 nt; and (iii) 10 aptamer–small molecule complexes,^79–87^ ranging from 25 to 55 nt. These categories ensured coverage of both single-stranded DNA and RNA aptamers, as well as diverse targets and binding modes. AlphaFold 3 was released in May 2024 and was trained on structures uploaded to the PDB before September 20, 2021. Of the 45 aptamer structures analyzed here, 37 (82%) were uploaded before this cutoff date. In addition, 6 well-characterized aptamers not represented in the PDB were selected based on their experimental validation in the literature by multiple independent studies. Collectively, this separate set of non-PDB aptamers serves as critical benchmarks for evaluating the merits of predictive models in the absence of reported structural coordinates.

### Structure Prediction Using AlphaFold 3 Performance Metrics

For each aptamer, including those lacking corresponding PDB structures, computational structure predictions were performed using AlphaFold 3 (DeepMind, London, UK) via the AlphaFold server (https://alphafoldserver.com). Default software parameters and model configurations were employed. The highest-ranked AlphaFold predictions, determined by its own internal confidence metrics, were selected for analysis. When initial predictions yielded poorly aligned aptamer structures, alternative seeds were tested to generate the most reliable model. Specifically, the predicted template modeling (pTM) score was used to assess the confidence of single-chain aptamer predictions, while the interface pTM (ipTM) score evaluated the reliability of complex structures, such as aptamer–target interactions. These two scores, ranging from 0 to 1, are referred to as chain pTM and ipTM, respectively, in the main text. The chain pTM score estimates the accuracy of a predicted structure relative to a true or theoretical model, while the ipTM score assesses the confidence in interchain interactions, highlighting regions where target-binding moieties are likely to occur. Where relevant, separate pTM scores for the target alone and the entire aptamer–target complex are also reported. These additional scores are referred to as the “target pTM” and “complex pTM”, respectively, in the main text. To streamline the collection of these metrics, a custom Python script was implemented that parses AlphaFold-generated JSON files to extract key indicators (ipTM, pTM, ranking score, fraction disordered, and additional interface-related data) and compile them into a CSV file. For pTM analysis, scores were categorized as high (≥0.7), moderate (0.5–0.7), or low (<0.5). As a comparative analysis of confidence metrics, the predicted local distance difference template (pLDDT) value, ranging from 1 to 100, was also considered. In contrast to assessing global structure confidence reflected in pTM values, the pLDDT value is a per-residue measure of prediction confidence. For pLDDT analysis, scores were categorized as high (≥70), moderate (50–70), or low (<50).

### Structural Analysis and Validation

Predicted and experimentally determined structures were analyzed in PyMOL (version 2.1, Schrödinger, LLC, New York, NY). Pairwise root-mean-square deviation (RMSD) values were calculated over backbone heavy atoms (P, C4, C3, and C2 for nucleic acids; central carbon, or C_α_ for proteins) to quantify structural similarity. Domains of particular interest, such as ligand-binding pockets and aptamer–protein interfaces, were visually inspected and aligned to confirm the fidelity of predictions. To further evaluate the accuracy of AlphaFold relative to PDB structural data, the Matthews correlation coefficient (MCC) was introduced as an additional metric.^88,89^ MCC is defined as

$$MCC=\frac{TP\times TN-FP\times FN}{\sqrt{\left(TP+FP\right)\left(TP+FN\right)\left(TN+FP\right)\left(TN+FN\right)}}$$

where TP, TN, FP, and FN represent the number of true-positive, true-negative, false-positive, and false-negative base pair predictions, respectively. Separate MCC scores were calculated for three interaction categories: (i) canonical base pairs, (ii) noncanonical base pairs, and (iii) base stacking. The secondary structure annotations were retrieved using the MC-Annotate tool hosted by the RNApdbee 2.0 web server (http://rnapdbee.cs.put.poznan.pl/) for both PDB-resolved aptamer structures and AlphaFold-predicted aptamer structures. A custom Python script then compared the interactions and computed MCC, precision, recall, F1 score, and accuracy for each interaction type. Additionally, noncanonical interaction categories were further subdivided into specific Leontis–Westhof interactions to count the base edge orientation (*cis* or *trans*) and subtype (Watson–Crick (W), Hoogsteen (H), or sugar (S)) involved in base pair interactions. A second custom Python script parsed the semicolon-delimited CSV outputs from MC-Annotate to record whether each aptamer structure contained any instance of a particular interaction subtype. These additional performance metrics provided a comprehensive and nuanced evaluation of how AlphaFold reconstructs structural features crucial for aptamer function.

All scripts used for nucleic acid interaction analysis are available in a GitHub repository (https://github.com/Sochoa8/nucleobase_interaction_calculator_annotated_NA_structures/tree/main).

## Supplementary Material



## Notes

Binary classification metrics including all MCC scores (Table S1); AlphaFold internal metrics for each predicted aptamer structure (Table S2); plot of aptamer per-nucleotide pLDDT scores as a function of RMSD values between AlphaFold predicted and PDB structures (Figure S1); structures of the *M08s-1* aptamer-thrombin complex (Figure S2); apo structures and CD spectroscopy of two streptavidin aptamers, *St-2–1* and *SteptApt5* (Figure S3); structures and CD spectroscopy of *S14* (Figure S4) and *Apt02* (Figure S5) G-quadruplex aptamers (PDF)

## Abbreviations

H: Hoogsteen
ipTM: interfacial predicted template modeling
MCC: Matthews correlation coefficient
pLDDT: predicted local distance difference template
pTM: predicted template modeling
RMSD: root-mean-square deviation
S: sugar
SELEX: systematic evolution of ligands by exponential enrichment
W: Watson
