# Supplementary material for: Direct Modeling of DNA and RNA Aptamers with AlphaFold 3: A Promising Tool for Predicting Aptamer Structures and Aptamer–Target Interactions
Source: ACS Synth Biol. 2025 Jul 3;14(8):3049–64. doi: 10.1021/acssynbio.5c00196 (PMC12362623; doi:10.1021/acssynbio.5c00196)
Supplement: Supplementary file 1 [file sb5c00196_si_001.pdf]

Supporting Information

Direct Modeling of DNA and RNA Aptamers with AlphaFold 3: A Promising Tool for Predicting Aptamer Structures and Aptamer-Target Interactions

Steven Ochoa[1], Valeria Milam[1,2,*]

Author Affiliations:

[1]School of Materials Science and Engineering
[2]Parker H. Petit Institute for Bioengineering and Bioscience,
Georgia Institute of Technology, 771 Ferst Dr. NW, Atlanta, GA 30332-0245, USA

Corresponding Author*:

Email: valeria.milam@mse.gatech.edu

Table S1. Summary of binary classification metrics for evaluating aptamer structure predictions. The table presents Matthews correlation coefficient (MCC), precision, recall, F1-score, and accuracy for canonical, noncanonical, and base stacking interaction predictions across all PDB structures surveyed in this work.

structure	MCC canonical	Precision canonical	Recall canonical	F1 canonical	Accuracy canonical	MCC noncanonical	Precision noncanonical	Recall noncanonical	F1 noncanonical	Accuracy noncanonical	MCC stacking	Precision stacking	Recall stacking	F1 stacking	Accuracy stacking
7zqs	0.00	0.00	0.00	0.00	0.99	-0.02	0.00	0.00	0.00	0.95	0.32	0.31	0.41	0.35	0.92
2l5k	1.00	1.00	1.00	1.00	1.00	-0.02	0.00	0.00	0.00	0.96	0.71	0.71	0.75	0.73	0.96
5hto	1.00	1.00	1.00	1.00	1.00	-0.01	0.00	0.00	0.00	0.97	0.41	0.44	0.42	0.43	0.95
4m6d	0.61	0.45	0.83	0.59	0.99	-0.01	0.00	0.00	0.00	0.98	0.23	0.25	0.27	0.26	0.94
8hba	0.88	0.85	0.92	0.88	0.99	-0.01	0.00	0.00	0.00	0.98	0.47	0.47	0.50	0.49	0.97
5d3g	0.91	0.83	1.00	0.91	1.00	0.00	0.00	0.00	0.00	0.99	0.71	0.66	0.79	0.72	0.98
5e54	0.95	1.00	0.90	0.95	1.00	0.00	0.00	0.00	0.00	0.99	0.52	0.52	0.55	0.53	0.98
7d7w	1.00	1.00	1.00	1.00	1.00	0.00	0.00	0.00	0.00	1.00	0.60	0.61	0.61	0.61	0.97
2qbz	0.46	0.44	0.49	0.46	1.00	0.05	0.06	0.04	0.05	1.00	0.52	0.50	0.56	0.53	0.99
8bw5	0.58	0.33	1.00	0.50	1.00	0.25	0.31	0.24	0.27	0.97	0.66	0.73	0.61	0.67	0.98
7d81	0.91	0.82	1.00	0.90	1.00	0.29	0.50	0.17	0.25	1.00	0.95	0.91	1.00	0.95	1.00
7zj5	0.98	0.98	0.99	0.98	1.00	0.31	0.38	0.26	0.31	1.00	0.84	0.83	0.85	0.84	1.00
6wlq	0.99	0.98	1.00	0.99	1.00	0.35	0.33	0.38	0.35	1.00	0.77	0.70	0.86	0.77	0.99
6wlk	0.97	0.98	0.96	0.97	1.00	0.38	0.40	0.36	0.38	1.00	0.85	0.77	0.94	0.85	1.00
2au4	0.91	0.83	1.00	0.91	1.00	0.38	0.50	0.30	0.38	0.99	0.64	0.55	0.77	0.64	0.98
8d29	0.55	0.50	0.63	0.56	0.99	0.41	0.50	0.33	0.40	0.99	0.76	0.73	0.83	0.77	0.98
1raw	1.00	1.00	1.00	1.00	1.00	0.41	0.33	0.50	0.40	1.00	0.89	0.86	0.93	0.89	0.99
8y0f	0.18	0.20	0.17	0.18	0.99	0.41	0.40	0.44	0.42	0.99	0.71	0.64	0.82	0.72	0.98
4pdb	0.96	1.00	0.93	0.96	1.00	0.44	0.50	0.40	0.44	0.99	0.90	0.85	0.97	0.91	0.99
3egz	0.94	0.88	1.00	0.94	1.00	0.49	0.67	0.36	0.47	1.00	0.85	0.82	0.88	0.85	0.99
2jwv	0.95	0.90	1.00	0.95	1.00	0.50	0.50	0.50	0.50	1.00	0.85	0.81	0.91	0.86	0.98
6k84	0.00	0.00	0.00	0.00	1.00	0.51	0.69	0.41	0.51	0.94	0.37	0.50	0.31	0.38	0.95
1ull	0.91	0.91	0.91	0.91	1.00	0.54	0.60	0.50	0.55	0.99	0.90	0.81	1.00	0.90	0.99
6wjs	0.87	0.76	1.00	0.86	1.00	0.61	0.54	0.70	0.61	1.00	0.84	0.78	0.91	0.84	1.00
1aw0	1.00	1.00	1.00	1.00	1.00	0.62	1.00	0.40	0.57	0.98	0.89	0.90	0.90	0.90	0.98
3zh2	0.81	0.67	1.00	0.80	0.99	0.63	0.50	0.80	0.62	0.99	0.77	0.70	0.89	0.78	0.97
3hxo	0.91	0.83	1.00	0.91	1.00	0.67	0.75	0.60	0.67	1.00	0.81	0.76	0.88	0.81	0.99
5kh8	1.00	1.00	1.00	1.00	1.00	0.71	1.00	0.50	0.67	1.00	0.79	0.77	0.83	0.80	0.99
7kd1	1.00	1.00	1.00	1.00	1.00	0.75	0.88	0.64	0.74	1.00	0.88	0.83	0.94	0.88	0.99
8gzk	1.00	1.00	1.00	1.00	1.00	0.75	0.75	0.75	0.75	0.99	0.63	0.58	0.73	0.65	0.96
3q51	1.00	1.00	1.00	1.00	1.00	0.77	1.00	0.60	0.75	0.99	0.94	0.92	0.96	0.94	0.99
4i7y	1.00	1.00	1.00	1.00	1.00	0.80	0.73	0.89	0.80	0.99	0.97	1.00	0.95	0.97	1.00
4m4o	0.97	1.00	0.95	0.97	1.00	0.80	0.86	0.75	0.80	1.00	0.92	0.96	0.90	0.93	1.00
2rqj	0.00	0.00	0.00	0.00	1.00	0.82	1.00	0.70	0.82	0.95	0.78	0.80	0.80	0.80	0.97
5lwj	1.00	1.00	1.00	1.00	1.00	0.82	1.00	0.67	0.80	1.00	0.91	0.86	0.96	0.91	0.99
7xhd	0.00	0.00	0.00	0.00	1.00	0.83	0.89	0.80	0.84	0.98	0.94	0.89	1.00	0.94	0.99
1ooa	0.81	0.67	1.00	0.80	0.99	0.86	1.00	0.75	0.86	1.00	0.82	0.80	0.87	0.83	0.98
8tfd	1.00	1.00	1.00	1.00	1.00	0.89	1.00	0.80	0.89	0.99	0.81	0.90	0.75	0.82	0.98
148d	0.89	1.00	0.80	0.89	0.99	0.94	0.89	1.00	0.94	0.99	1.00	1.00	1.00	1.00	1.00
4dih	0.00	0.00	0.00	0.00	1.00	0.94	1.00	0.89	0.94	0.99	0.81	0.78	0.88	0.82	0.97
1aw4	1.00	1.00	1.00	1.00	1.00	0.94	1.00	0.89	0.94	1.00	0.89	0.94	0.85	0.89	0.99
5cmx	1.00	1.00	1.00	1.00	1.00	1.00	1.00	1.00	1.00	1.00	0.91	0.87	0.95	0.91	0.99
6evv	1.00	1.00	1.00	1.00	1.00	1.00	1.00	1.00	1.00	1.00	0.94	0.89	1.00	0.94	0.99
6tf1	1.00	1.00	1.00	1.00	1.00	1.00	1.00	1.00	1.00	1.00	0.91	0.86	0.97	0.91	0.99
6u82	1.00	1.00	1.00	1.00	1.00	1.00	1.00	1.00	1.00	1.00	0.91	0.84	1.00	0.91	0.99
7e5p	0.00	0.00	0.00	0.00	1.00	1.00	1.00	1.00	1.00	1.00	1.00	1.00	1.00	1.00	1.00

Table S2. Summary of the internal confidence metrics output by AlphaFold for each aptamer structure analyzed in this study. Metrics include average pLDDT (predicted local distance difference template), pTM (predicted Template Modeling score), ipTM (inter-residue predicted TM-score), and other statistical confidence values used to assess the reliability of AlphaFold's structural predictions.

PDB ID/Name	Avg. pLDDT	iPTM	pTM	Ranking Score	Number of Recycles	Fraction Disordered	Chain Pair iPTM	Chain Pair PAE Min	Chain PTM
5E54	72.15		0.41	0.41	10	0	[[0.41]]	[[0.84]]	[0.41]
148D	70.41		0.01	0.01	10	0	[[0.01]]	[[0.91]]	[0.01]
1AM0	90.81	0.68	0.68	0.68	10	0	[[0.32, 0.68], [0.68, 0.55]]	[[0.77, 2.09], [1.46, 0.8]]	[0.32, 0.55]
1AW4	84.48	0.57	0.6	0.57	10	0	[[0.29, 0.22, 0.47], [0.22, 0.3, 0.46], [0.47, 0.46, 0.29]]	[[0.77, 5.7, 4.64], [5.92, 0.77, 4.24], [2.57, 2.58, 0.82]]	[0.29, 0.3, 0.29]
1OOA	78.32	0.87	0.75	0.86	10	0.03	[[0.74, 0.87], [0.87, 0.36]]	[[0.76, 1.47], [1.35, 0.79]]	[0.74, 0.36]
1RAW	91.11	0.66	0.62	0.65	10	0	[[0.33, 0.66], [0.66, 0.44]]	[[0.77, 2.03], [1.47, 0.8]]	[0.33, 0.44]
1ULL	91.66	0.58	0.61	1.08	10	1	[[0.03, 0.58], [0.58, 0.45]]	[[0.76, 1.53], [1.52, 0.8]]	[0.03, 0.45]
2AU4	86.48	0.65	0.66	0.65	10	0	[[0.49, 0.65], [0.65, 0.49]]	[[0.77, 2.57], [1.81, 0.81]]	[0.49, 0.49]
2JWV	83.73		0.25	0.25	10	0	[[0.25]]	[[0.82]]	[0.25]
2L5K	74.81		0.18	0.18	10	0	[[0.18]]	[[0.88]]	[0.18]
2QBZ	67.47		0.49	0.49	10	0	[[0.49]]	[[0.82]]	[0.49]
2RQJ	84.14		0.01	0.01	10	0	[[0.01]]	[[0.88]]	[0.01]
3HXO	67.43	0.7	0.87	0.74	10	0	[[0.9, 0.7], [0.7, 0.43]]	[[0.76, 2.06], [2.73, 0.83]]	[0.9, 0.43]
3Q51	89.67		0.52	0.52	10	0	[[0.52]]	[[0.79]]	[0.52]
3ZH2	23.45	0.94	0.95	0.94	10	0	[[0.94, 0.95, 0.94, 0.93, 0.23], [0.95, 0.94, 0.94, 0.94, 0.23], [0.94, 0.94, 0.94, 0.95, 0.25], [0.93, 0.94, 0.95, 0.94, 0.25], [0.23, 0.23, 0.25, 0.25, 0.14]]	[[0.76, 0.85, 0.87, 0.97, 16.33], [0.85, 0.76, 0.97, 0.87, 16.29], [0.87, 0.98, 0.76, 0.85, 15.78], [1.01, 0.89, 0.85, 0.76, 15.33], [25.8, 25.29, 19.99, 20.02, 0.85]]	[0.94, 0.94, 0.94, 0.94, 0.14]
4DIH	84.63	0.78	0.86	0.85	10	0.12	[[0.41, 0.83, 0.29], [0.83, 0.86, 0.71], [0.29, 0.71, 0.01]]	[[0.76, 1.42, 5.29], [1.17, 0.76, 3.84], [8.94, 4.52, 0.84]]	[0.41, 0.86, 0.01]
4I7Y	82.93	0.9	0.93	0.96	10	0.12	[[0.55, 0.9, 0.33, 0.48], [0.9, 0.93, 0.87, 0.88], [0.33, 0.87, None, 0.43], [0.48, 0.88, 0.43, 0.31]]	[[0.76, 0.9, 4.02, 2.6], [0.8, 0.76, 2.93, 1.3], [None, None, None, None], [3.46, 1.23, 2.62, 0.79]]	[0.55, 0.93, None, 0.31]
4M4O	77.47	0.46	0.73	0.51	10	0	[[0.86, 0.46], [0.46, 0.5]]	[[0.76, 2.96], [3.04, 0.81]]	[0.86, 0.5]
4M6D	81.15	0.59	0.79	0.63	10	0	[[0.83, 0.59], [0.59, 0.47]]	[[0.76, 1.79], [2.15, 0.8]]	[0.83, 0.47]
4PDB	94.99	0.88	0.82	0.95	10	0.15	[[0.79, 0.88], [0.88, 0.57]]	[[0.76, 0.95], [0.99, 0.78]]	[0.79, 0.57]
5CMX	80.16	0.85	0.9	0.92	10	0.11	[[0.49, 0.87, 0.35, 0.49], [0.87, 0.91, 0.88, 0.81], [0.35, 0.88, None, 0.61], [0.49, 0.81, 0.61, 0.36]]	[[0.76, 1.05, 2.99, 2.07], [0.92, 0.76, 2.24, 1.46], [None, None, None, None], [3.51, 1.62, 1.3, 0.8]]	[0.49, 0.91, None, 0.36]
5D3G	85.7	0.92	0.91	0.93	10	0.02	[[0.9, 0.91, 0.91], [0.91, 0.87, 0.88], [0.91, 0.88, 0.49]]	[[0.76, 0.98, 1.52], [0.94, 0.76, 1.82], [1.36, 1.72, 0.78]]	[0.9, 0.87, 0.49]
5HTO	73.85	0.89	0.89	0.93	10	0.08	[[0.87, 0.89, 0.89], [0.89, 0.87, 0.88], [0.89, 0.88, 0.4]]	[[0.76, 0.84, 1.27], [0.83, 0.76, 1.66], [1.31, 2.23, 0.8]]	[0.87, 0.87, 0.4]
5KH8	69.99		0.37	0.37	10	0	[[0.37]]	[[0.84]]	[0.37]
5LWJ	59.74	0.73	0.73	0.73	10	0	[[0.57, 0.73], [0.73, 0.52]]	[[0.76, 0.98], [0.91, 0.78]]	[0.57, 0.52]
6EVV	94.09	0.92	0.94	0.98	10	0.11	[[0.59, 0.92, 0.52, 0.54, 0.64], [0.92, 0.95, 0.94, 0.96, 0.9], [0.52, 0.94, None, None, 0.53], [0.54, 0.96, None, None, 0.14], [0.64, 0.9, 0.53, 0.14, 0.34]]	[[0.76, 0.83, 1.77, 2.2, 1.27], [0.78, 0.76, 1.36, 1.27, 1.09], [None, None, None, None, None], [None, None, None, None, None], [1.62, 1.16, 1.07, 4.7, 0.78]]	[0.59, 0.95, None, None, 0.34]
6K84	63.34		0.18	0.18	10	0	[[0.18]]	[[0.86]]	[0.18]
6TF1	98.05	0.79	0.8	0.79	10	0	[[0.56, 0.17, 0.38, 0.27, 0.12, 0.09, 0.05, 0.07, 0.07, 0.88], [0.17, None, None, None, None, None, None, None, None, 0.51], [0.38, None, None, None, None, None, None, None, None, 0.71], [0.27, None, None, None, None, None, None, None, None, 0.57], [0.12, None, None, None, None, None, None, None, None, 0.36], [0.09, None, None, None, None, None, None, None, None, 0.33], [0.05, None, None, None, None, None, None, None, None, 0.16], [0.07, None, None, None, None, None, None, None, None, 0.4], [0.07, None, None, None, None, None, None, None, None, 0.22], [0.88, 0.51, 0.71, 0.57, 0.36, 0.33, 0.16, 0.4, 0.22, 0.71]]	[[0.76, 5.6, 1.72, 2.88, 5.41, 6.74, 11.41, 9.79, 13.0, 0.9], [None, None, None, None, None, None, None, None, None, None], [None, None, None, None, None, None, None, None, None, None], [None, None, None, None, None, None, None, None, None, None], [None, None, None, None, None, None, None, None, None, None], [None, None, None, None, None, None, None, None, None, None], [None, None, None, None, None, None, None, None, None, None], [None, None, None, None, None, None, None, None, None, None], [None, None, None, None, None, None, None, None, None, None], [0.85, 4.06, 1.62, 2.75, 4.83, 5.5, 10.28, 7.65, 11.47, 0.77]]	[0.56, None, None, None, None, None, None, None, None, 0.71]
6U82	93.21	0.89	0.91	0.94	10	0.09	[[0.9, 0.89], [0.89, 0.61]]	[[0.76, 1.23], [1.01, 0.77]]	[0.9, 0.61]
6WJS	83.18		0.64	0.64	10	0	[[0.64]]	[[0.8]]	[0.64]
6WLK	67.62		0.45	0.45	10	0	[[0.45]]	[[0.81]]	[0.45]
6WLQ	69.38		0.53	0.53	10	0	[[0.53]]	[[0.81]]	[0.53]
7D7W	97.8	0.75	0.79	0.76	10	0	[[0.65, 0.56, 0.47, 0.39, 0.32, 0.23, 0.18, 0.13, 0.19, 0.11, 0.1, 0.02, 0.82], [0.56, None, None, None, None, None, None, None, None, None, None, None, 0.67], [0.47, None, None, None, None, None, None, None, None, None, None, None, 0.56], [0.39, None, None, None, None, None, None, None, None, None, None, None, 0.47], [0.32, None, None, None, None, None, None, None, None, None, None, None, 0.4], [0.23, None, None, None, None, None, None, None, None, None, None, None, 0.43], [0.18, None, None, None, None, None, None, None, None, None, None, None, 0.25], [0.13, None, None, None, None, None, None, None, None, None, None, None, 0.17], [0.19, None, None, None, None, None, None, None, None, None, None, None, 0.45], [0.11, None, None, None, None, None, None, None, None, None, None, None, 0.19], [0.1, None, None, None, None, None, None, None, None, None, None, None, 0.15], [0.02, None, None, None, None, None, None, None, None, None, None, None, 0.04], [0.82, 0.67, 0.56, 0.47, 0.4, 0.43, 0.25, 0.17, 0.45, 0.19, 0.15, 0.04, 0.71]]	[[0.76, 2.06, 2.88, 3.9, 4.33, 6.54, 7.19, 9.98, 7.25, 10.31, 12.24, 23.28, 0.93], [None, None, None, None, None, None, None, None, None, None, None, None, None], [None, None, None, None, None, None, None, None, None, None, None, None, None], [None, None, None, None, None, None, None, None, None, None, None, None, None], [None, None, None, None, None, None, None, None, None, None, None, None, None], [None, None, None, None, None, None, None, None, None, None, None, None, None], [None, None, None, None, None, None, None, None, None, None, None, None, None], [None, None, None, None, None, None, None, None, None, None, None, None, None], [None, None, None, None, None, None, None, None, None, None, None, None, None], [None, None, None, None, None, None, None, None, None, None, None, None, None], [None, None, None, None, None, None, None, None, None, None, None, None, None], [None, None, None, None, None, None, None, None, None, None, None, None, None], [0.82, 1.78, 2.56, 3.67, 3.95, 4.5, 6.46, 9.27, 4.31, 9.0, 10.47, 22.27, 0.78]]	[0.65, None, None, None, None, None, None, None, None, None, None, None, 0.71]
7D81	87.71	0.76	0.74	0.76	10	0	[[0.61, 0.51, 0.39, 0.12, 0.77], [0.51, None, None, None, 0.61], [0.39, None, None, None, 0.48], [0.12, None, None, None, 0.21], [0.77, 0.61, 0.48, 0.21, 0.58]]	[[0.76, 2.44, 4.2, 12.27, 1.08], [None, None, None, None, None], [None, None, None, None, None], [None, None, None, None, None], [0.94, 2.1, 3.64, 10.62, 0.8]]	[0.61, None, None, None, 0.58]
7E5P	88.71		0.02	0.02	10	0	[[0.02]]	[[0.79]]	[0.02]
7KD1	67.11		0.44	0.44	10	0	[[0.44]]	[[0.83]]	[0.44]
7XHD	90.62	0.62	0.64	0.63	10	0	[[None, None, None, None, None, 0.04, 0.03], [None, None, None, None, None, 0.04, 0.03], [None, None, None, None, None, 0.03, 0.04], [None, None, None, None, None, 0.02, 0.04], [None, None, None, None, None, 0.03, 0.03], [0.04, 0.04, 0.03, 0.02, 0.03, 0.03, 0.52], [0.03, 0.03, 0.04, 0.04, 0.03, 0.52, 0.03]]	[[None, None, None, None, None, None, None], [None, None, None, None, None, None, None], [None, None, None, None, None, None, None], [None, None, None, None, None, None, None], [None, None, None, None, None, None, None], [1.14, 1.05, 1.32, 1.7, 1.37, 0.77, 1.17], [1.64, 1.34, 1.07, 1.25, 1.36, 1.18, 0.77]]	[None, None, None, None, None, 0.03, 0.03]
7ZJ5	43.91		0.23	0.23	10	0	[[0.23]]	[[0.82]]	[0.23]
7ZQS	52.85	0.25	0.8	0.44	10	0.16	[[0.83, 0.25], [0.25, 0.16]]	[[0.76, 10.23], [13.8, 0.86]]	[0.83, 0.16]
8BW5	41.83	0.6	0.76	0.69	10	0.1	[[0.32, 0.77, 0.06], [0.77, 0.82, 0.21], [0.06, 0.21, 0.2]]	[[0.76, 1.99, 15.46], [1.42, 0.76, 13.39], [23.31, 18.87, 0.85]]	[0.32, 0.82, 0.2]
8D29	85.63		0.34	0.34	10	0	[[0.34]]	[[0.82]]	[0.34]
8GZK	73.74	0.15	0.18	0.15	10	0	[[None, 0.15], [0.15, 0.17]]	[[None, None], [5.67, 0.87]]	[None, 0.17]
8HBA	43.09	0.32	0.37	0.33	10	0	[[0.4, 0.1, 0.33], [0.1, 0.41, 0.28], [0.33, 0.28, 0.29]]	[[0.78, 13.64, 8.18], [12.73, 0.78, 9.91], [6.0, 9.51, 0.84]]	[0.4, 0.41, 0.29]
8TFD	80.04	0.64	0.85	0.68	10	0	[[0.87, 0.64], [0.64, 0.05]]	[[0.76, 2.23], [2.75, 0.87]]	[0.87, 0.05]
8Y0F	77.26		0.3	0.3	10	0	[[0.3]]	[[0.87]]	[0.3]

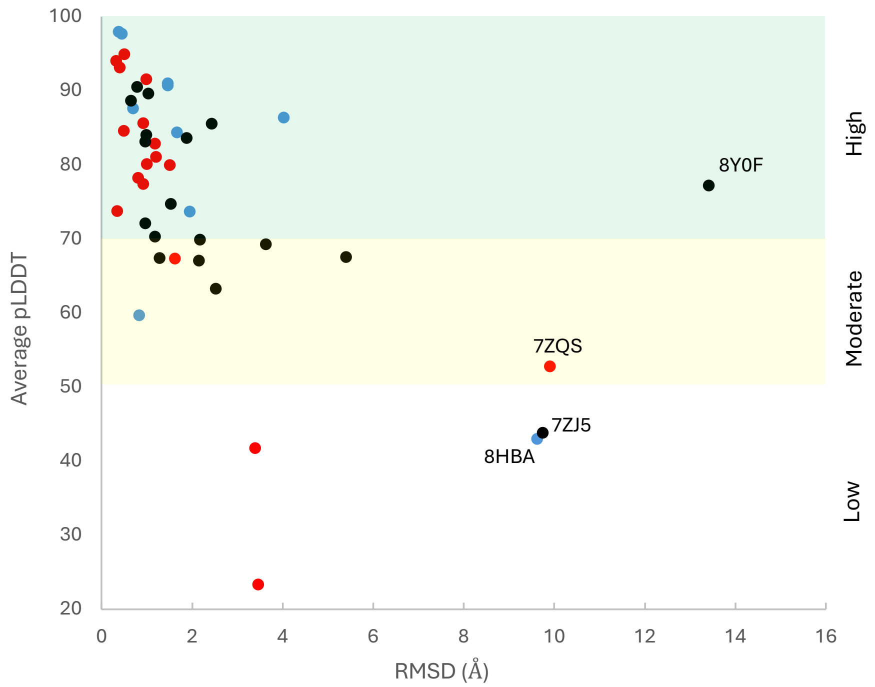

Figure S1. Average pLDDT scores plotted as a function of RMSD values between AlphaFold predicted and PDB structures.

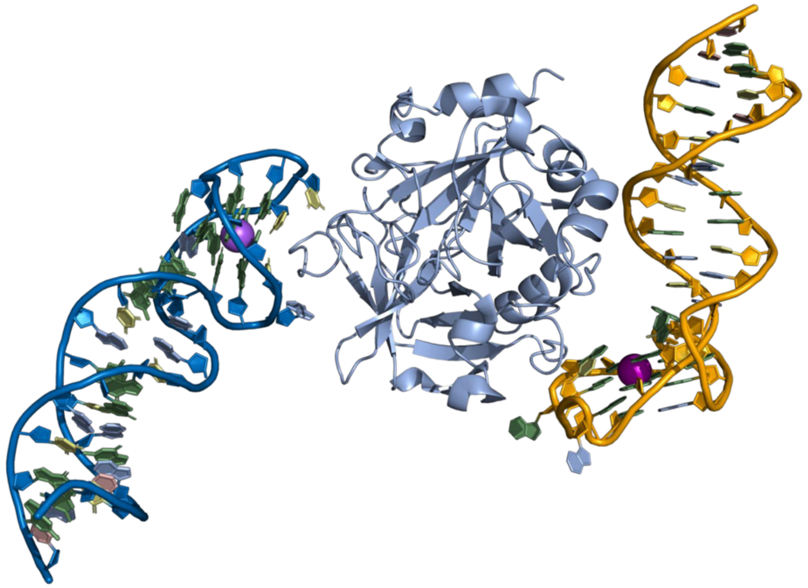

Figure S2. PDB structure (8BW5) of *M08s-1* DNA aptamer reported by Troisi et al.[69] (blue backbone) in complex with thrombin (light blue). AlphaFold predicted complex superimposed by aligning the protein backbone. AlphaFold predicted *M08s-1* aptamer structure (orange backbone) bound to thrombin C-terminus.

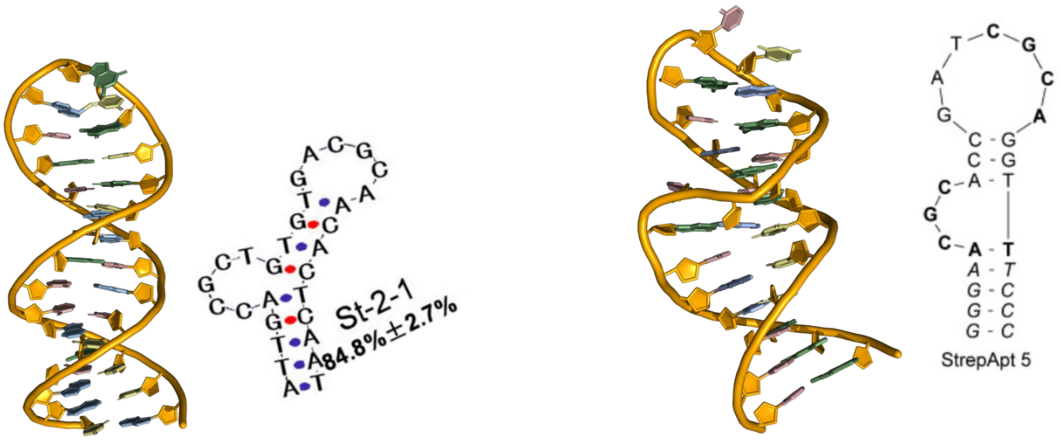

Figure S3. AlphaFold predictions of apo structures of *St-2-1* (left) and *StreptApt5* (right) aptamers compared with mFold predictions reported, respectively, by Bing et al.[38] and Ruigrok et al. [103]

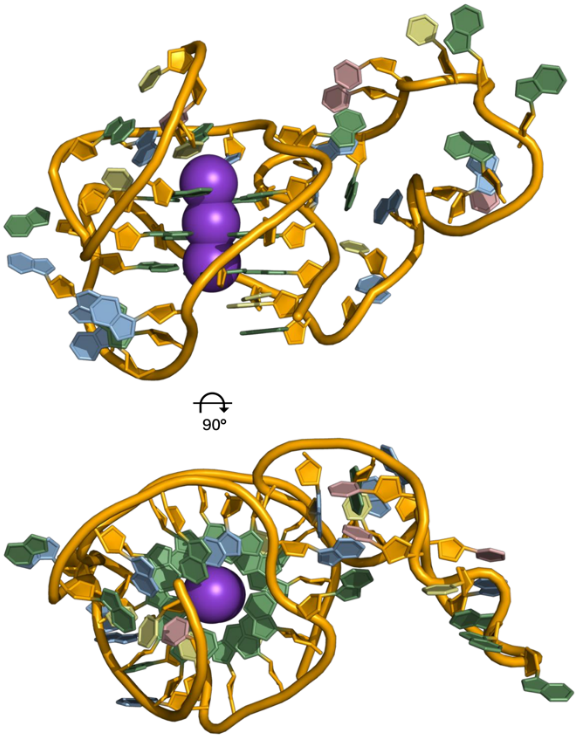

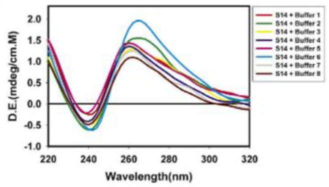

aptamer	QGRS sequence	G-Score
S14	GGGACATAGTGGGGAAAGAGGGGAAGAGTGGG	71

Figure S4. AlphaFold predicted structure of *S14* aptamer demonstrating parallel G-quadruplex. Experimental data presented by Gupta et al.[104] illustrating CD measurements using buffers of various potassium concentrations in addition to QGRS mapper results.

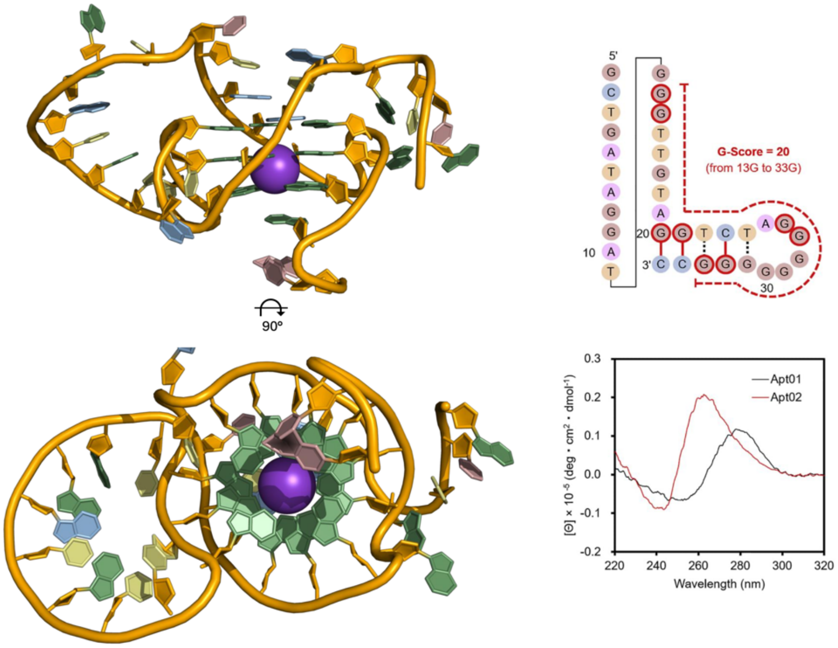

Figure S5. AlphaFold prediction of *Apt02*, identified through SELEX by Yoshitomi et al.[105] QGRS mapper predictions CD measurements showing a negative peak at ~240 nm and positive peak ~260 nm, suggesting *Apt02* folds into a parallel G-quadruplex conformation.
